# Cinnamaldehyde derivatives act as antimicrobial agents against *Acinetobacter baumannii* through the inhibition of cell division

**DOI:** 10.3389/fmicb.2022.967949

**Published:** 2022-08-29

**Authors:** Wern Chern Chai, Jonathan J. Whittall, Steven W. Polyak, Klyie Foo, Xin Li, Cameron J. Dutschke, Abiodun D. Ogunniyi, Shutao Ma, Matthew J. Sykes, Susan J. Semple, Henrietta Venter

**Affiliations:** ^1^Health and Biomedical Innovation, Clinical and Health Sciences, University of South Australia, Adelaide, SA, Australia; ^2^Department of Medicinal Chemistry, Key Laboratory of Chemical Biology (Ministry of Education), School of Pharmaceutical Sciences, Cheeloo College of Medicine, Shandong University, Jinan, China; ^3^Australian Centre for Antimicrobial Resistance Ecology, School of Animal and Veterinary Sciences, University of Adelaide, Adelaide, SA, Australia; ^4^Quality Use of Medicines and Pharmacy Research Centre, Clinical and Health Sciences, University of South Australia, Adelaide, SA, Australia

**Keywords:** antimicrobial resistance, antimicrobial drug development, FtsZ, FtsZ inhibitor, cinnamaldehyde, gram-negative, XDR *Acinetobacter baumannii*

## Abstract

*Acinetobacter baumannii* is a pathogen with high intrinsic antimicrobial resistance while multidrug resistant (MDR) and extensively drug resistant (XDR) strains of this pathogen are emerging. Treatment options for infections by these strains are very limited, hence new therapies are urgently needed. The bacterial cell division protein, FtsZ, is a promising drug target for the development of novel antimicrobial agents. We have previously reported limited activity of cinnamaldehyde analogs against *Escherichia coli*. In this study, we have determined the antimicrobial activity of six cinnamaldehyde analogs for antimicrobial activity against *A. baumannii*. Microscopic analysis was performed to determine if the compounds inhibit cell division. The on-target effect of the compounds was assessed by analyzing their effect on polymerization and on the GTPase activity of purified FtsZ from *A. baumannii*. *In silico* docking was used to assess the binding of cinnamaldehyde analogs. Finally, *in vivo* and *in vitro* safety assays were performed. All six compounds displayed antibacterial activity against the critical priority pathogen *A. baumannii*, with 4-bromophenyl-substituted **4** displaying the most potent antimicrobial activity (MIC 32 μg/mL). Bioactivity was significantly increased in the presence of an efflux pump inhibitor for *A. baumannii* ATCC 19606 (up to 32-fold) and significantly, for extensively drug resistant UW 5075 (greater than 4-fold), suggesting that efflux contributes to the intrinsic resistance of *A. baumannii* against these agents. The compounds inhibited cell division in *A. baumannii* as observed by the elongated phenotype and targeted the FtsZ protein as seen from the inhibition of polymerization and GTPase activity. *In silico* docking predicted that the compounds bind in the interdomain cleft adjacent to the H7 core helix. Di-chlorinated **6** was devoid of hemolytic activity and cytotoxicity against mammalian cells *in vitro*, as well as adverse activity in a *Caenorhabditis elegans* nematode model *in vivo*. Together, these findings present halogenated analogs **4** and **6** as promising candidates for further development as antimicrobial agents aimed at combating *A. baumannii*. This is also the first report of FtsZ-targeting compounds with activity against an XDR *A. baumannii* strain.

## Introduction

*Acinetobacter baumannii* has been recognized as one of the most difficult to treat resistant Gram-negative bacterial pathogens (Knight et al., 2018; Abdi et al., 2020; Kyriakidis et al., 2021; Murray et al., 2022). This microorganism is intrinsically resistant to multiple distinct classes of antibiotics (Knight et al., 2018; Abdi et al., 2020). The rise of antimicrobial resistance (AMR) is further compounding the issue (Ambrose et al., 2022), making infections caused by *A. baumannii* more challenging and expensive to address. Consequently, the World Health Organization (WHO) has prioritized *A. baumannii* as one of the most critical pathogens in urgent need of new antimicrobial drug discovery and development (World Health Organization, 2017). In line with the WHO Global Action Plan, one of the objectives is to increase the development of new chemotherapies with novel mechanisms of action to combat multidrug resistant pathogens (World Health Organization, 2015), especially *A. baumannii*.

A promising route toward the development of novel antimicrobial agents necessary to combat AMR is to inhibit bacterial cell division. FtsZ is a key protein that is crucial in cellular division in both Gram-positive and Gram-negative bacteria and is therefore a promising antibacterial drug target (Hurley et al., 2016; Carro, 2019; Kusuma et al., 2019; Casiraghi et al., 2020). During cellular division, FtsZ functions in concert with 11 other essential proteins to form a complex known as the divisome (Bisson-Filho et al., 2017). The divisome is necessary for the formation of two daughter cells by first forming a dynamic ring (Z ring) around the bacterial midcell that ultimately contracts, leading to closure of the septum (Broughton et al., 2015; Bisson-Filho et al., 2017; den Blaauwen and Luirink, 2019). Self-polymerization of FtsZ in a GTP-dependent mechanism is critical to the formation of the Z-ring structure. Given its promise as an antibacterial drug target, multiple classes of small molecules that bind to FtsZ have been identified and reported in the literature (Margalit et al., 2004; Haydon et al., 2008; Kapoor and Panda, 2009; Chan et al., 2013; Kaul et al., 2015; Li and Ma, 2015; Liu et al., 2018; Carro, 2019; Kusuma et al., 2019; Casiraghi et al., 2020; Chai et al., 2020). These include the benzamides (Haydon et al., 2008; Kaul et al., 2015; Bi et al., 2017, 2018; Chai et al., 2020; Straniero et al., 2020); coumarins (de Souza et al., 2005; Yang et al., 2021); berberine derivatives (Domadia et al., 2008); quinazolines and quinolinium compounds (Nair et al., 2017; Sun et al., 2018a,b; Zheng et al., 2018); cinnamaldehydes (Domadia et al., 2007; Li et al., 2015a), zantrins (Margalit et al., 2004), methyl-phenyl-phenanthridium derivatives (Liu et al., 2018) and quinuclidine derivatives (Chan et al., 2013). Whilst several FtsZ inhibitors have efficacy against Gram-positive bacteria, very few display antimicrobial activity against Gram-negative bacteria (Araújo-Bazán et al., 2016; Casiraghi et al., 2020; Gurnani et al., 2022; Rosado-Lugo et al., 2022; Zhong et al., 2022), leaving the development of FtsZ inhibitors with activity against pathogens such as *A. baumannii* as an area in need of urgent exploration.

The FtsZ protein comprises two globular subdomains, with the GTP-binding site located in the N-terminal domain and a GTPase activating site at the C-terminal end. These subdomains are separated by the central H7 helix (Löwe and Amos, 1998; Matsui et al., 2012). Whilst the N-terminal is generally well conserved between bacterial species, the flexible C-terminal subdomain differs in both length and structure (Huecas et al., 2017). Crystal structures of *S. aureus* FtsZ in complex with PC190723 and other benzamides have defined the mode of binding for these inhibitors (Matsui et al., 2012; Ferrer-González et al., 2017; Fujita et al., 2017). Since the FtsZ protein plays an essential role in cellular division of prokaryotes (Romberg et al., 2001; Busiek and Margolin, 2015; Aylett and Duggin, 2017), its function is comparable to mammalian tubulin in eukaryotes (Pilhofer et al., 2007; McIntosh et al., 2010; Sundararajan et al., 2015; Battaje and Panda, 2017). However, bacterial FtsZ has distinctive structural differences from mammalian tubulin that permit the development of bacterial-specific inhibitors. Primary structure analysis has revealed the proteins are distinct, with FtsZ from *Escherichia coli* and human tubulin sharing only 10% homology (Chen and Erickson, 2008). The crystal structures also reveal important differences, for example that the interdomain cleft in FtsZ necessary for inhibitor binding is not observed in tubulin (Kusuma et al., 2019). These key features allow for development of new antibacterial agents that specifically target FtsZ.

**FIGURE 1:**
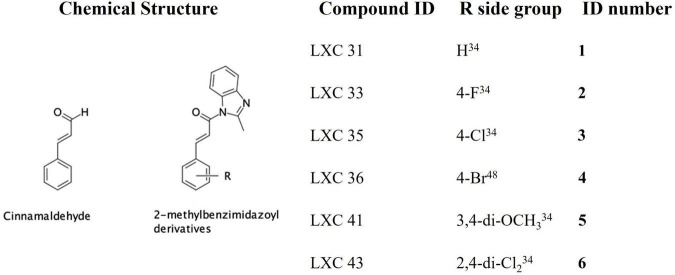
Chemical structures of cinnamaldehyde and analogs.

The natural product cinnamaldehyde (Figure 1) is one promising lead compound with modest bioactivity against *E. coli* (MIC 1 mg/mL) (Domadia et al., 2007; Li et al., 2015a). *E. coli* treated with cinnamaldehyde produces a characteristic enlarged and elongated rod-shaped morphology consistent with the disruption of the divisome and arrest of cell division (Domadia et al., 2007). Furthermore, biochemical analysis focused upon *E. coli* FtsZ has revealed that cinnamaldehyde inhibited GTPase activity (IC_50_ 5.8 μM) as well as self-polymerization (ED_50_ 6.9 μM) (Domadia et al., 2007). Docking studies have proposed the molecular basis of inhibition is *via* cinnamaldehyde binding buttressed against the H7 helix, in a binding mechanism analogous to that observed for the benzamides, with the aldehyde group directed into the central cavity of the protein and the benzyl moiety interacting with the T7 loop (Domadia et al., 2007). This structural data suggested that more potent cinnamaldehyde derivatives could be realized through chemical modification of the aldehyde and benzyl moieties. Due to its promising bioactivity toward Gram-negative pathogens, we commenced chemical optimization of cinnamaldehyde with the objective of developing more potent analogs with broad spectrum antimicrobial activity (Li et al., 2015a,b). Indeed, new compounds with superior antibacterial efficacy compared to cinnamaldehyde were produced by appending a methylbenzimidazolyl moiety onto the aldehyde (see compound **1**, Figure 1) with modest antimicrobial activity against both *E. coli* ATCC 25922 and *Pseudomonas aeruginosa* ATCC 27853 (MIC 128 μg/mL). A library of new analogs was subsequently generated by substitution of the aromatic ring with halogen or di-methoxy moieties. The most potent exemplars from the chemical optimization studies are shown in Figure 1 (see compounds **2** to **6**) (Li et al., 2015a,b).

In this study, we sought to further investigate the biological properties of promising candidates **1** to **6** (the LXC series, Figure 1) against a broad panel of Gram-positive and Gram-negative bacteria, including the ESKAPE pathogens (Rice, 2008). Detailed microbiological and biochemical studies were performed to understand the effect of these compounds on the clinically important and critical priority intrinsically resistant pathogen, *A. baumannii* (World Health Organization, 2017). These studies demonstrated the efficacy of the compounds in targeting growth inhibition and cell division arrest, and confirmed *in vitro* activity was indeed through the inhibition of FtsZ from *A. baumannii*. This is the first study to report the cloning of *A. baumannii* FtsZ and development of assays required for biochemical characterization of FtsZ inhibitors against this important pathogen. Off-target effects were addressed including activity against mammalian tubulin and potential cytotoxicity using mammalian cell lines and the nematode *Caenorhabditis elegans*.

## Materials and methods

### Chemicals

All chemicals were obtained from Sigma or Chem-Supply unless otherwise indicated.

Derivatives of cinnamaldehyde **1** to **6** were synthesized, chemically characterized, and assessed for purity, as described previously (Li et al., 2015a,b). Compounds were initially dissolved in DMSO. For the biological assays, a final DMSO concentration of 2.5% (v/v) was used unless otherwise specified.

### Bacterial strains and growth conditions

Reference bacterial strains were obtained from the American Type Culture Collection (ATCC). Vancomycin resistant *Enterococcus faecium* (VRE 734, wastewater isolate from the Venter Laboratory Collection) was isolated on Bile Esculin agar (Oxoid, Australia) containing 6 μg/mL vancomycin and identified using MALDI-TOF mass spectrometry (Bruker, Preston, Victoria, Australia, Australian Center for Antimicrobial Resistance Ecology). The *Pseudomonas aeruginosa* WT PAO1 and *Escherichia coli* with deletions of both *acrA* and *acrB* genes encoding drug efflux pumps were obtained from the Venter laboratory stock (Welch et al., 2010). The protocol for microbial growth was adopted from the European Committee on Antimicrobial Susceptibility Testing (EUCAST) (European Committee for Antimicrobial Susceptibility Testing, 2016).

Non-fastidious bacteria and clinical isolates were maintained in Mueller Hinton (MH) broth (Acumedia, United Kingdom) supplemented with 15% (v/v) glycerol (Chem-Supply, Australia), cultured on Mueller Hinton (MH) agar (Acumedia, United Kingdom) plated and incubated at 37°C. Fastidious bacteria were maintained in Brain Heart Infusion (BHI) broth (Acumedia, United Kingdom) supplemented with 15% (v/v) glycerol at −80°C. The fastidious bacteria were cultured on MH agar plates supplemented with 5% (v/v) lysed horse blood and 3% (v/v) fetal bovine serum and incubated at 30°C in the presence of 5% CO_2_. Drug susceptibility assays were carried out with the non-fastidious cultures grown in cation-adjusted Mueller Hinton (CaMH) broth (Becton Dickinson, France). *Streptococcus pyogenes* was grown in CaMH broth supplemented with 5% (v/v) lysed horse blood and 3% (v/v) fetal bovine serum whereas *Enterococcus faecium* was grown in BHI broth.

### Antimicrobial susceptibility assays

The minimum inhibitory concentrations (MICs) for all six compounds were determined using the microbroth dilution protocol as per EUCAST with an inoculum of approximately 5 × 10^6^ CFU/mL in an exponential growth phase (European Committee for Antimicrobial Susceptibility Testing, 2016). Bacterial suspension, 100 μL/well, was added to 100 μL/well of drug dilutions, then incubated for 18 h at 37°C. Fastidious bacterial suspensions were instead incubated at 30°C in the presence of 5% CO_2_. Bacterial growth was assessed by measuring the change in A_600_ nm between time 0 and 18 h (Cytation5^®^ Cell Imaging Multi-Mode Reader, Bio-Tek^®^). Levofloxacin was used as the positive control. Assays were completed in duplicate in at least three independent experiments.

### Inhibition of efflux pump using phenylalanine arginine-β-naphthylamide

To determine if expression of efflux pumps would play a role in the efflux of the cinnamaldehyde derivatives leading to poor antimicrobial activity, interactions between PAβN and the LXC compounds were assessed by checkerboard titration assay. The checkerboard titration assay was performed as previously described (Chai et al., 2020). Briefly, PAβN was added in the first row and serially diluted in CaMH broth along the ordinate of the microwell plate while the LXC compounds were serially diluted along the abscissa. Bacterial suspension for *A. baumannii* ATCC 19606 or AB UW 5075 100 μL/well were added as described for the MIC assay. Bacterial growth was assessed at A_600_ nm between time 0 and 18 h (Cytation5^®^ Cell Imaging Multi-Mode Reader, Bio-Tek^®^).

### Time-kill assays against *Acinetobacter baumannii*

To determine if the LXC compounds would have bactericidal or bacteriostatic effects, the growth inhibition kinetics were analyzed using the assay as previously described (Chai et al., 2020) with the following modifications. LXC compounds were tested at 1×, 2×, or 4× MIC against *A. baumannii* ATCC 19606. The bacterial suspension of approximately 1.5 × 10^8^ CFU/mL was prepared in a 1.5 mL Eppendorf^®^ tube. An aliquot was then serially diluted into a microtiter plate before being spot plated onto a fresh MH agar plate. The tube was incubated, and the process was repeated at 1, 3, 6, and 18 h post-exposure to the compounds. All MH agar plates were incubated at 37°C for 18 h. Colonies were counted to calculate the CFU/mL. Dilutions were prepared to ensure the count was kept between 2 and 20 colonies per spot. Levofloxacin was used as a control at sub-MIC (0.25 μg/mL) and 4 × MIC (2 μg/mL) concentrations to produce bacteriostatic and bactericidal effects, respectively. The results from each drug concentration were obtained from at least three independent experiments with different batches of cells.

### Determination of cell division phenotype

Morphological changes of bacteria when exposed to the compounds were assessed microscopically using the assay as previously described (Chai et al., 2020). Eppendorf^®^ tubes were set up with *A. baumannii* ATCC 19606 as described for the time-kill assays. At time 0, 1, 3, 6, and 18 h, an aliquot of the bacterial suspension (1 μL) was transferred onto a glass slide and viewed under a light microscope (Olympus CX33^®^) at 100 × magnification. Representative images were captured using JENOPTIK GRYPHAX^®^ imaging.

### Cloning of *Acinetobacter baumannii* ATCC 19606 FtsZ protein

The gene encoding *Ab*FtsZ was obtained *via* PCR amplification from genomic DNA of *A. baumannii* ATCC 19606 using forward 5′-AGGGTTTCATATGTTAGAATT TGAACAAGGATTTAATC-3′ and reverse 5′-AGGGTTTCTC GAGACGTCTTGGTTCTTCTTGAACG-3′ oligonucleotides containing *Nde*I and *Xho*I sites respectively (underlined). The amplicon was then ligated into *Nde*I and *Xho*I sites treated pET-41a(+) (Novagen) vector. The resulting 8-His tagged FtsZ construct was confirmed by Sanger sequencing. This plasmid was transformed into *E. coli* DH5α™ silver (Bioline) for amplification then subsequently into *E. coli* BL21(DE3) for recombinant protein production.

### Over-expression and purification of the *Ab*FtsZ

Bacterial culture was initially grown to logarithmic phase in prewarmed Luria Bertani (Becton Dickinson, France) broth at 37°C for 5 h before cooling to 25°C. *Ab*FtsZ expression was subsequently induced by the addition of 1 mM isopropyl-β-D-1-thiogalactopyronoside (IPTG; Thermo-Fisher, Australia) for 18 h at 25°C. The cells were harvested by centrifugation at 5,000 *g* for 20 min and then resuspended in Buffer A (50 mM Tris HCl pH 8.0, 200 mM NaCl and 10% (w/v) glycerol) supplemented with cOmplete™, EDTA-free Protease Inhibitor Cocktail (Roche) and 20 μg/mL DNAse (Sigma, Australia) before lysis using a cell disruptor (Constant Systems E1061, Thermo Scientific, Australia) at 30 kPsi. The lysate was clarified by ultra-centrifugation (Optima XPN-100 Ultracentrifuge, Beckman Colter, Australia) at 200,000 *g* for 45 min. *Ab*FtsZ was then purified from the supernatant using 2 × 1 mL Histrap HP columns (GE Healthcare) on an Äkta FPLC (GE Healthcare). Lysate was loaded onto the column pre-equilibrated in Buffer A, washed for 10 column volumes with Buffer A containing 20 mM imidazole then eluted with Buffer A at 150 mM imidazole. Fractions containing the desired protein were identified by SDS-PAGE (4–12% NuPAGE Bis-Tris polyacrylamide gels (Invitrogen, Australia). Those fractions containing pure *Ab*FtsZ were pooled and exchanged into Buffer A using a HiTrap desalting column (GE Healthcare) to remove imidazole. Aliquots of the purified FtsZ were snap frozen in liquid nitrogen and stored at −80°C.

### Protein concentration determination

The protein concentration was measured using the BioRad™ BCA Protein Assay Standard Kit, according to the manufacturer’s instructions with bovine serum albumin used as a standard. The A_750_ nm was measured using a Cytation5^®^ Cell Imaging Multi-Mode Reader (Bio-Tek^®^).

### GTPase assay

Cinnamaldehyde analogs that inhibited the FtsZ-catalyzed hydrolysis of GTP were assessed using a malachite green-phosphomolybdate colorimetric assay previously described (Chai et al., 2020) with the following modification. The reactions were carried out at 37°C using 0.1 mg/mL of purified *Ab*FtsZ which was mixed with the compounds at a defined concentration in GTP reaction buffer (50 mM Tris HCl pH 7.2, 300 mM KCl and 5 mM MgCl_2_).

### Effects on *Ab*FtsZ polymerization using 90° light scattering

Effects on inhibition of the cinnamaldehyde derivatives on *Ab*FtsZ polymerization were analyzed using the assay as previously described (Chai et al., 2020) with the following modifications. A reaction mix was prepared in a cold Eppendorf^®^ tube where *Ab*FtsZ (0.15 mg/mL) was mixed with the test compound at the desired concentration and a polymerization buffer diluted from a double strength buffer to give a final concentration of 25 mM PIPES pH 6.8, 50 mM KCl and 10 mM MgCl_2_. The reaction mix was prepared such that the DMSO concentration remained constant at 2% (v/v). The mix was then added into a quartz cuvette (PerkinElmer^®^, United Kingdom), and placed in a PerkinElmer^®^ LS 55 fluorometer under the fixed condition of excitation/emission at 350/350 nm, slit width < 2 nm, a 1 s read interval. The temperature was calibrated to remain constant at 8°C. The fluorescence was followed for 300 sec to allow equilibration, then polymerization was initiated with the addition of 1 mM GTP. A separate negative control was prepared where 1 mM GDP was added instead of the GTP. The reaction was followed for 600 s.

### Computational studies

The chemical structures of compounds **1** to **6** were generated using PICTO (version 4.5.1.0, OpenEye Scientific Software) then saved as SMILES strings before conversion into 3-dimensional conformers (maximum of 200 conformers per compound) *via* Omega (version 4.1.0.0, OpenEye Scientific Software) using default settings (Hawkins et al., 2010). Four *Staphylococcus aureus* FtsZ (*Sa*FtsZ) co-complex crystal structures with bound benzamide inhibitors (PDB ID: 3VOB, 5XDT, 5XDU and 6KVP) (Matsui et al., 2012; Ferrer-González et al., 2017; Fujita et al., 2017) served as receptors. The protein receptor structures were prepared using Make Receptor (version 3.0.1, OpenEye Scientific Software), with the *bona fide* benzamide binding pocket defined as the target binding site. *In silico* docking of the multi-conformer database into each receptor was performed using the FRED module in OEDocking (version 3.2.0.2, OpenEye Scientific Software) with default settings (McGann, 2011, 2012; Kelley et al., 2015). The docking results were ranked by the Chemgauss 4 scoring function, with the best scoring conformer of each compound retained. An additional 9 poses were subsequently investigated for each molecule by using the FRED flag num_poses. Binding poses were visualized using VIDA (version 4.4.0.4, OpenEye Scientific Software).

### Mammalian tubulin polymerization assay

The effects of the cinnamaldehyde analogs were analyzed for off-target effects on mammalian tubulin using the protocol as previously described (Chai et al., 2020), using a Tubulin Polymerization Assay Kit (Cytoskeleton Inc., BK011P, Denver, CO, United States), following the protocol described by the manufacturer.

LXC compounds were tested at 1 × MIC with the final concentration of 2.5% (v/v) DMSO maintained in the assay. Controls included 20 μM paclitaxel (promoter of polymerization), 20 μM vinblastine (inhibitor of polymerization) and 2.5% (v/v) DMSO (solvent control). The reaction was initiated by the addition of 1 mM GTP at 37°C. The fluorescence was read for 2,000 s using PerkinElmer Enspire^®^ plate reader.

### Cytotoxicity analysis of LXC compounds on mammalian cells

The effects of the cinnamaldehyde analogs were analyzed for off-target effects on mammalian cells using the protocol as previously described (Chai et al., 2020) with the following modifications. *In vitro* cytotoxicity was assessed in HepG2 (ATCC HB-8065) cells using the RealTime-Glo™ MT Cell Viability Assay Kit (Promega) as described previously (Wang et al., 2017). The LXC compounds were tested at 1 × and 2 × MIC using a final DMSO concentration of 2% (v/v) in the assay. Two controls, 2% (v/v) DMSO and 50 μg/mL ampicillin were used. The luminescence signal was read at 5-min intervals over 24 h in a Cytation5^®^ Cell Imaging Multi-Mode Reader (Bio-Tek^®^), at 37°C in the presence of 5% CO_2_.

The hemolysis assay was performed using fresh human red blood cells (RBCs). PBS solution (137 mM NaCl, 2.7 mM KCl, 1.46 mM KH_2_PO_4_, 8.1 mM NaH_2_PO_4_ pH 7.4) was used to wash the RBCs three times at 500 *g* for 5 min, then resuspended in 1% (w/v) PBS solution. Compounds (8 μL) were added to a 96-microwell plate, which were serially diluted from 4 × MIC in 2% (v/v) DMSO. A 1% (v/v) Triton X-100, 2% (v/v) DMSO and 128 μg/mL ampicillin were used as controls. These were performed in quadruplicate. Thereafter, 192 μL RBCs were added into all wells and the plates were incubated at 37°C under constant shaking (100 rpm) for 1 h. RBCs were precipitated by centrifugation of the plates (1,000× *g* for 3 min). An aliquot (100 μL) of each supernatant was transferred into a new 96-microwell plate and the A_450_ nm was determined using a PerkinElmer Enspire^®^ plate reader. The fraction of intact RBC for each sample was determined as fraction of the intact RBCs for the sample without the addition of compounds (set at 100%) and plotted as a function of compound concentration.

### Cytotoxicity analysis of LXC compounds on *Caenorhabditis elegans* nematodes

Protocols for growing, harvesting, and synchronizing *C. elegans* have been previously described (Porta-de-la-Riva et al., 2012; Chai et al., 2020) and were performed with the following modifications. The nematodes were treated with LXC compounds **1** to **6** at 1 × and 2 × MIC with at least 3 replicates using a final DMSO concentration of 2% (v/v). The plate was incubated at 25°C and the number of live vs dead nematodes was enumerated every 24 h for 72 h. Separate wells containing 50 μg/mL ampicillin and 2% (v/v) DMSO were used as controls. The percentage of living nematodes at each time point across 72 h was calculated to give the percentage of survival.

## Results and discussion

### The LXC compounds display antimicrobial activity against *Acinetobacter baumannii*

Compounds **1** to **6** were examined for antimicrobial activity against an extended panel of Gram-positive and Gram-negative bacteria that included isolates of the clinically important ESKAPE pathogens and drug-resistant strains. Critically for this study, *A. baumannii* ATCC 19606 was the only Gram-negative microorganism broadly susceptible to all of the LXC compounds with the MIC values ranging from 32 to 256 μg/mL (Table 1). Noteworthy were the brominated analog **4** (MIC 32 μg/mL) that had similar potency to gentamicin, and the di-chlorinated analog **6** (MIC 64 μg/mL). All compounds were similarly assayed against the extensively drug resistant (XDR) *A. baumannii* UW 5075. However, none of the LXC compounds displayed any antibacterial activity against this XDR strain (Table 1). Similarly, no antibacterial activity was observed against other Gram-negative bacteria in the panel, including *Klebsiella pneumoniae* ATCC 4352, ATCC 13883 and ATCC 33495 and *Pseudomonas aeruginosa* PAO1 and ATCC 27853. Weak antimicrobial activity was observed against *Streptococcus pneumoniae* ATCC 6303 (MIC 256 μg/mL for all compounds), but MRSA ATCC 43300 was only susceptible to **6** (MIC 64 μg/mL) (Supplementary Table 1). Due to the urgent and unmet need to develop new antimicrobial agents that target *A. baumannii* (World Health Organization, 2017), subsequent studies were focused upon this important critical priority Gram-negative bacterium.

**TABLE 1:** Minimum Inhibitory Concentration (MIC) of the LXC compounds and common antimicrobial agents on two strains of *Acinetobacter baumannii*.

Compounds			MIC (μg/mL)	
ID	Name	R side chain	*A. baumannii* ATCC 19606	*A. baumannii* XDR UW 5075
**1**	LXC 31	H	128	>128
**2**	LXC 33	4-F	128	>128
**3**	LXC 35	4-Cl	128	>128
**4**	LXC 36	4-Br	32	>128
**5**	LXC 41	3,4-di-OCH_3_	256	>128
**6**	LXC 43	2,4-di-Cl	64	>128
	Amoxycillin		64	1024
	Gentamicin		16	>1024
	Imipenem		1	128
	Levofloxacin		0.5	8
	Colistin		2	2

### Time-kill curves for LXC compounds indicate a bacteriostatic mechanism of action

Time-kill studies with *A. baumannii* ATCC 19606 were conducted to determine if Compounds **1** to **6** were bactericidal or bacteriostatic in their mechanism of action. In all cases, the cell numbers remained constant across 18 h suggesting the compounds are bacteriostatic in their mechanism of action (Supplementary Figure 1). Previous studies have suggested that cinnamaldehyde is bacteriostatic on *E. coli* (Domadia et al., 2007) however there are no other studies examining the effects of cinnamaldehyde or its derivatives on *A. baumannii*.

Although *A. baumannii* is intrinsically more resistant to multiple antibiotics, and much more resistant than other pathogens such as *E. coli*, we observed higher efficacy for the LXC compounds against *A. baumannii* compared to *E. coli* and *P. aeruginosa*. We have previously observed a similar phenomenon where robenidine only displayed antimicrobial activity against *A. baumannii*, but not other Gram-negative pathogens (Khazandi et al., 2019). The outer membrane of Gram-negative pathogens acts as a permeability barrier that prevents xenobiotic compounds from penetrating into the bacterial cell (Arzanlou et al., 2017). The outer membrane of *A. baumannii* is about 100 times more impermeable than that of *E. coli* as *A. baumannii* lacks general, non-specific trimeric porins found in *E. coli* (Nikaido, 2003; Zgurskaya et al., 2015). However, due to discreet differences in the lipid A component, *A. baumannii* tends to be more permeable to amphiphilic molecules compared to *E. coli* (Krishnamoorthy et al., 2017).

### Active efflux plays a role in the resistance to the LXC compounds

Poor permeation across cell membranes and active drug efflux of antimicrobial agents are well-known intrinsic resistance mechanisms employed by bacteria (Miki and Hardt, 2013; Venter et al., 2015; Arzanlou et al., 2017; Knight et al., 2018). Hence, we investigated if the absence of whole cell activity noted above for **1** to **6** against the *A. baumannii* XDR strain could be explained by either of these factors. Antimicrobial susceptibility assays were repeated following permeabilization of the bacterial outer membrane with either EDTA or colistin (Alakomi et al., 2006; Farrag et al., 2019; Khazandi et al., 2019). Neither treatment changed the MIC values (data not shown) indicating that the outer membrane barrier was not responsible for limiting the efficacy of the LXC compounds. Next, the antimicrobial sensitivity assays were performed in the presence of the well-characterized drug efflux pump inhibitor, PAβN (Sharma et al., 2019). The potency of all six compounds against *A. baumannii* ATCC 19606 was improved when assayed in combination with the efflux pump inhibitor PAβN, with the MIC decreasing to as low as 8 μg/mL for compounds **3** to **6** (Table 2). Importantly, the presence of PAβN bestowed significantly improved antimicrobial activity to the LXC compounds against the XDR strain UW 5075 (Table 2). Noteworthy were compounds **5** and **6** that were inactive alone but displayed an MIC of 32 μg/mL when assayed in combination with PAβN. The improved antimicrobial activities were observed at concentrations of PAβN that did not damage the outer membrane (Supplementary Figure 2) implying that efflux was, indeed, a major factor responsible for impaired activity of the LXC compounds.

**TABLE 2:** MIC of the LXC compounds against *A. baumannii* ATCC 19606 and XDR UW 5075 in the absence or presence of PAβN.

		MIC, μg/mL							

**Compounds**		***A. baumannii* ATCC 19606**				***A. baumannii* XDR UW 5075**			
		**+PAβN, μg/mL**				**+PAβN, μg/mL**			
		**0**	**50**	**100**	**200**	**0**	**50**	**100**	**200**
**1**	LXC 31	128	128	64	32	>128	>128	128	64
**2**	LXC 33	128	128	32	16	>128	>128	64	64
**3**	LXC 35	128	128	8	8	>128	>128	128	64
**4**	LXC 36	32	32	8	8	>128	>128	128	64
**5**	LXC 41	256	128	8	8	>128	>128	64	32
**6**	LXC 43	64	32	16	8	>128	>128	32	32

### Cinnamaldehyde analogs disrupt cellular division

**FIGURE 2:**
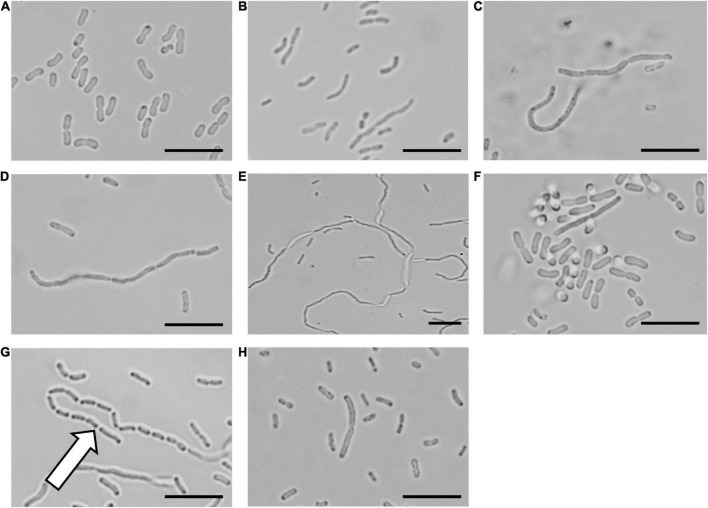
The LXC compounds inhibit cell division in *Acinetobacter baumannii* ATCC 19606 *A. baumannii* ATCC 19606 culture was adjusted to a density of approximately 2.7 × 10^8^ CFU/mL in MH broth and incubated at 37°C with the LXC compounds. A 1 μL aliquot was taken out at varying timepoints (0, 1, 3, 6, and 18 h) and imaged by light microscopy. The images for 6 h are depicted here. Images are **(A)** in the absence of the compounds, **(B)** with the divisome inhibitor, divin at 64 μg/mL, or with the LXC compounds at 2 × MIC values: **(C)** compound **1** at 256 μg/mL, **(D)** compound **2** at 256 μg/mL, **(E)** compound **3** at 256 μg/mL, **(F)** compound **4** at 64 μg/mL, **(G)** compound **5** at 512 μg/mL and **(H)** compound **6** at 128 μg/mL. The white arrow indicates “grooving” site, an indication of where separation of daughter cells should take place. Scale bar is 50 μm.

To confirm that the cinnamaldehyde derivatives disrupted cellular division, microscopy analysis was performed on *A. baumannii* ATCC 19606 treated with the LXC compounds (Figure 2). The morphology of the bacterium was observed using light microscopy at varying time points. The bacterium was also treated with the non-specific cell division inhibitor divin, which yielded the elongated cell shape characteristic of a rod-shaped bacterium unable to divide (Figures 2A,B). When treated with the LXC compounds at 2 × MIC, all six analogs induced the expected elongated phenotype for *A. baumannii* within 6 h (Figures 2C–H). Interestingly, this same morphological change was readily visible within 3 h post-treatment for halogenated compounds **2**, **3**, **4**, and **6** but not the unsubstituted compound **1**, suggesting that modification of the benzyl ring of cinnamaldehyde was important for increased whole cell activity (Supplementary Figure 3). An expanded version of the microscopy data with all the timepoints and concentrations tested is provided in Supplementary Figure 3. Interestingly, “grooving” (shown with white arrow in Figure 2G) between cells was observed. This is consistent with daughter cells that have undergone division but are unable to separate. Grooving has previously been reported in other rod-shaped bacteria including *B. subtilis*, *E. coli* and *P. aeruginosa*, when cellular division was arrested (Anderson et al., 2004; de Boer, 2010; Annapurna et al., 2015; Pichoff et al., 2018; Araya et al., 2019; Sharavanan et al., 2019). Complementary time-kill studies were also performed upon *A. baumannii* ATCC 19606 (Supplementary Figure 1). The number of viable cells remained constant throughout the 18 h time-course, indicative of a bacteriostatic mechanism of action. The absence of bactericidal activity was consistent with the lack of cell lysis noted in the microscopy analysis above. Together, these data are consistent with the mechanism of antibacterial action being through the inhibition of cell division.

Previously, we have shown that cinnamaldehyde analogs inhibit the cellular division of *E. coli* and *P. aeruginosa*, creating an elongated phenotype (Li et al., 2015a,b). Here, we observed the same morphological change in *A. baumannii*. Inhibition of *A. baumannii* creating this effect is also a feature of the FtsZ targeting compounds. Although this assay is often used as a screening tool to identify FtsZ inhibitors, the on-target effects of these LXC compounds with the FtsZ protein from *A. baumannii* (*Ab*FtsZ) have not been reported. Therefore, efficacy of these LXC compounds on purified *Ab*FtsZ *in vitro* had to be verified.

### Cloning and preparation of recombinant FtsZ from *Acinetobacter baumannii* ATCC 19606

**FIGURE 3:**
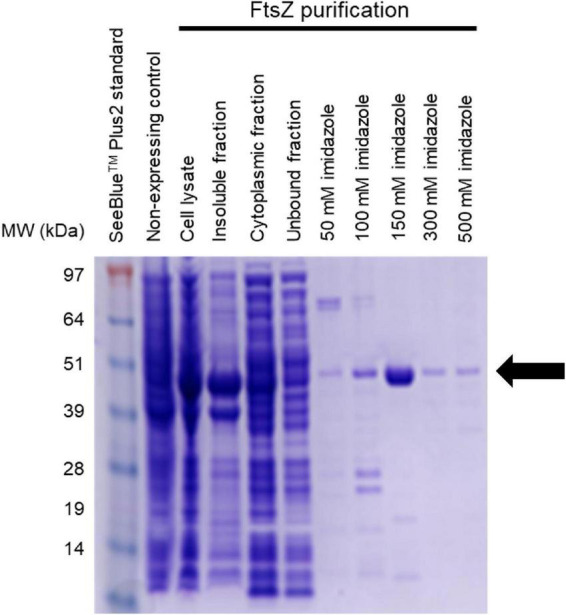
Purification of *Ab*FtsZ *Ab*FtsZ was cloned in the pET-41a(+) vector and expressed in BL21(DE3) *E. coli via* induction with 1 mM IPTG. The protein was purified from the cytoplasmic fraction using Nickel-affinity column chromatography. The different fractions from the purification process were loaded onto SDS-PAGE (4–12%). Protein was visualized by staining with Coomassie^®^ Brilliant Blue R-250. Purified *Ab*FtsZ (43.4 kDa) is observed as indicated by the arrow. The protein concentration was determined using the standard Bio-Rad™ BCA Protein Assay Kit.

To confirm that the mechanism of action of the LXC compounds was indeed through inhibition of FtsZ, further biochemical studies were performed. The *ftsZ* gene was obtained by PCR using genomic DNA isolated from *A. baumannii* ATCC 19606 and ligated in the pET-41a(+) vector for over-expression in *E. coli*. An 8-His tag was fused onto the C-terminus to facilitate purification by immobilized metal ion chromatography (Figure 3). Conditions for culturing and expression of *Ab*FtsZ in *E. coli* BL21 (DE3) were optimized for high level production of FtsZ that could be purified to homogeneity in a single chromatography step, with an approximate yield of 20 mg of pure *Ab*FtsZ per liter culture. This protein was subsequently used for *in vitro* biochemical assays to characterize the cinnamaldehyde analogs.

### Cinnamaldehyde analogs inhibit *Ab*FtsZ GTPase activity

**FIGURE 4:**
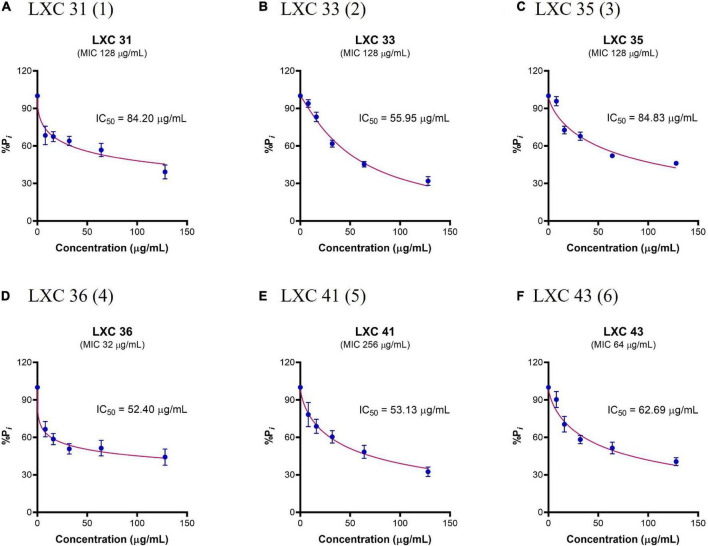
The LXC compounds inhibit GTPase activity in a dose-dependent manner. **(A–F)** The GTPase activity of purified *Ab*FtsZ was determined from the liberation of P*_*i*_* from GTP using malachite green. *Ab*FtsZ was incubated with the LXC compounds for 30 min before the reaction was initiated by addition of 1 mM GTP. The reaction was terminated by the addition of citric acid after 10 min. P*_*i*_* release rate was normalized to the sample without the addition of LXC compounds (86.26 μM P*_*i*_*/mg *Ab*FtsZ/min) that was taken as 100%. The results are presented in mean ± SEM. Statistical analysis was performed using one-way ANOVA (statistically significance, *p* < 0.05). A non-linear regression model was used in GraphPad Prism 8.3 to determine the IC_50_.

Purified *Ab*FtsZ protein was used to confirm that the LXC compounds exerted their bioactivity directly upon this target. As the hydrolysis of GTP accompanies FtsZ polymerization, the enzymatic activity of *Ab*FtsZ was measured by quantifying the liberation of inorganic phosphate. All six of the LXC compounds inhibit GTPase activity at the highest concentration (128 μg/mL, Figure 4) with IC_50_ values between 52 and 85 μg/mL. Interestingly, the IC_50_ of these LXC compounds does not correlate with their respective MIC values (Figure 4). The differences in the biochemical and whole cell activities are consistent with our earlier observation about the role of efflux pumps and the inability of the compounds to obtain suitable cytotoxic concentrations inside the bacteria necessary to inhibit the FtsZ target.

### Cinnamaldehyde analogs inhibit *Ab*FtsZ polymerization

**FIGURE 5:**
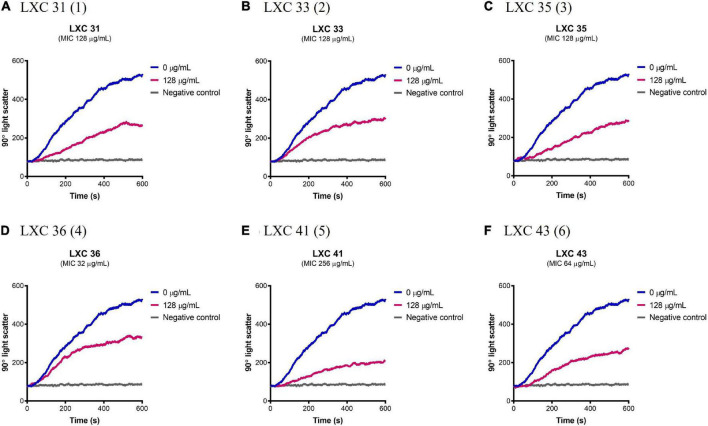
The LXC compounds inhibit *Ab*FtsZ polymerization. **(A–F)** *Ab*FtsZ (0.15 mg/mL) was pre-incubated in polymerization buffer (25 mM PIPES pH 6.8, 50 mM KCl and 10 mM MgCl_2_) together with the test compound at 128 μg/mL for 300 s. Polymerization was then initiated with the addition of 1 mM GTP. The fluorescence was measured as a function of time at excitation and emission wavelengths of A_350_ and A_350_ nm, respectively, and a slit width of <2 nm. The blue line in each panel indicates the addition of 2.5% (v/v) DMSO, the pink line indicates the test compound tested at 128 μg/mL and the gray line represents the addition of 1 mM GDP (as negative control) instead of GTP.

Having established that the LXC compounds inhibited GTPase activity, the effect of these compounds on polymerization of *Ab*FtsZ was directly measured using a 90° light scattering assay (Figure 5). This was performed using a fluorescence spectrometer, thermostatically controlled at 8°C, to measure the increased absorbance of scattered light at A_350_ nm that correlate with changes in protein polymerization. Here, FtsZ was preincubated with either the test compounds at 128 μg/mL (pink curves, Figure 5) or a vehicle control of 2.5% (v/v) DMSO (blue curves, Figure 5). Protein polymerization was then initiated by the addition of GTP. Consistent with earlier reports, polymerization activity was highly specific for GTP as the addition of 1 mM GDP resulted in no change in signal as FtsZ remained within its unpolymerized state (gray curves, Figure 5). All cinnamaldehyde derivatives inhibited protein polymerization to varying extents, with the strongest activity observed with compound **5**, followed by compounds **1** and **6** (*p* < 0.05). We have previously reported inhibition of FtsZ from *E. coli* (Li et al., 2015a,b) using this light scattering assay. In these previous studies, impaired polymerization was reported for compounds **2**, **3** and **6** at 120 μg/mL (Li et al., 2015a). Interestingly, compound **6** displayed a similarly potent inhibition on the polymerization of *Ab*FtsZ.

### *In silico* docking studies provide insight into the possible binding modes of the LXC compounds in the interdomain cleft of FtsZ

**FIGURE 6:**
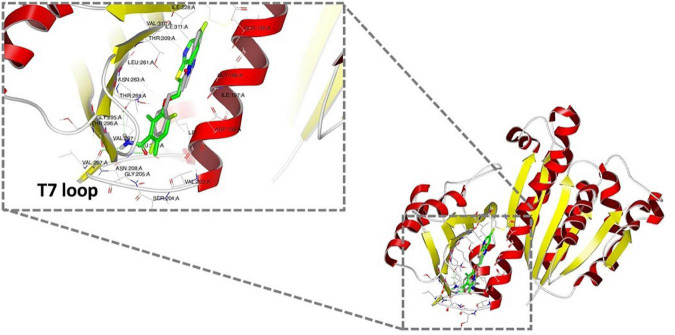
Docking analysis of the predicted binding of cinnamaldehyde analogs to FtsZ *In silico* docking studies were performed using OpenEye Scientific Software (McGann, 2012). Molecular binding of LXC 31 (6; gray) is shown above alongside co-crystallized FtsZ inhibitor PC190723 (green; PDB 3VOB) (Matsui et al., 2012). Binding poses for compounds **1** to **6** are shown in Supplementary Figure 4. In each case, the lowest energy pose of the lowest energy conformer is depicted.

As bioactive cinnamaldehyde analogs **1** to **6** (Li et al., 2015a,b) targeted *A. baumannii* specifically through inhibition of *Ab*FtsZ, we were interested to further investigate the potential interactions between the compounds and FtsZ. Docking studies of cinnamaldehyde have previously predicted binding in the interdomain cleft similar to the benzamides (Domadia et al., 2007). Molecular docking studies were performed to see if the cinnamaldehyde analogs were also likely to behave similarly. Crystal structures of FtsZ in complex with di-fluoro-benzamide analogs (Matsui et al., 2012; Ferrer-González et al., 2017; Fujita et al., 2017) were employed for *in silico* docking studies using the OEDocking module of OpenEye Scientific Software (McGann, 2012). The common inhibitor binding pocket identified in the co-complexes with the benzamides was selected to dock the cinnamaldehydes as the chemical structures of both classes share similar planar, elongated geometries. The docking results with PDB 3VOB (co-crystallized with benzamide PC190723) are shown here as an exemplar of the results obtained. PC190723 was firstly redocked, and the expected X-ray binding orientation was replicated. Subsequent docking analysis of compounds **1** to **6** showed that they were all able to be accommodated in the same inhibitor binding site and adopted binding poses consistent with the binding orientations observed with benzamides (Figure 6). The compounds all buttressed against central helix H7 with the substituted benzyl rings generally orientated toward the center of the protein in a pocket lined by Gln 184, Met 218 and Ile 220. At the other end of the compound, the common imidazolyl moiety was generally oriented toward the solvent exposed T7 loop for the lowest energy pose of each compound. The only exception to this was **6**, which was predicted to have an inverted orientation in its lowest energy pose. Analysis of further higher energy poses of **6**, showed a mixture of orientations. Compounds **1** to **6** all have an aromatic ring at both ends of their structures, which may give some flexibility in possible orientations within the binding pocket. It was also notable that **6** had the highest degree of shape similarity with PC190723 (data not shown). Analysis of the docking scores revealed that all six test compounds bound with affinities that were comparable with the *bona fide* benzamide ligands (Supplementary Figure 4 and Supplementary Table 2). Given that the molecular analysis supported the mechanism of action being through the inhibition of FtsZ, the biological properties of **1** to **6** were further investigated.

### Establishing the pre-clinical safety of cinnamaldehyde analogs

To investigate the potential of compounds **1** to **6** as preclinical candidates, a series of *in vitro* and *in vivo* assays were performed. Here, the aim was to identify those compounds that possess the desirable safety profile in candidates for future drug development. The effect of the compounds on the polymerization of mammalian tubulin was addressed to find those compounds with the necessary selectivity toward the bacterial target. The cytotoxicity of all compounds was then investigated against two mammalian cell lines before assaying for toxicity *in vivo* in a nematode model. Together, these data provide insight into the preclinical safety profile of our new cinnamaldehyde analogs.

### Selective binding toward bacterial FtsZ

**FIGURE 7:**
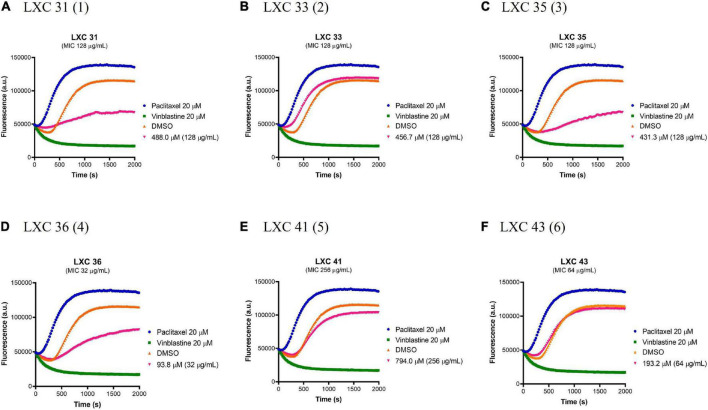
The effect of the LXC compounds on the polymerization of mammalian tubulin. **(A–F)** The polymerizing activity of the LXC compounds on porcine tubulin was assessed using a commercial Tubulin Polymerization Assay Kit. The LXC compounds at 1 × MIC were pre-incubated with tubulin on ice before the reaction was initiated by the addition of 1 mM GTP. The fluorescence was measured as a function of time at A_360_ and A_420_ nm excitation and emission wavelengths, respectively, at the constant temperature of 37°C. DMSO 2.5% (v/v) (brown curves) was used as vehicle control while paclitaxel (polymerization stabilizer; blue curves) and vinblastine (polymerization inhibitor; green curves) at 20 μM each were also included as controls.

Prokaryotic FtsZ is a structural homolog of eukaryotic tubulin. Consequently, it was essential to assess all preclinical candidates that target bacterial FtsZ for off-target binding to the mammalian tubulin. The effect of all six cinnamaldehyde derivatives on polymerization of human tubulin was analyzed using a commercial tubulin assay kit. The compounds were assayed alongside the known tubulin polymerization stimulator paclitaxel (blue curves, Figure 7) and inhibitor vinblastine (green curves, Figure 7). The compounds were tested at 1 × MIC (pink curves, Figure 7) and compared to a vehicle control of 2.5% (v/v) DMSO (brown curves, Figure 7). The non-substituted analog **1** showed undesirable inhibitory activity, as did mono-halogenated compounds **3** and **4**. In contrast, fluorinated **2** and di-substituted analogs **5** and **6** were devoid of tubulin polymerization activity even at concentrations ∼10–20 times higher than that of paclitaxel and vinblastine (Figure 7).

### Cytotoxicity of the cinnamaldehyde analogs against a mammalian cell line

**FIGURE 8:**
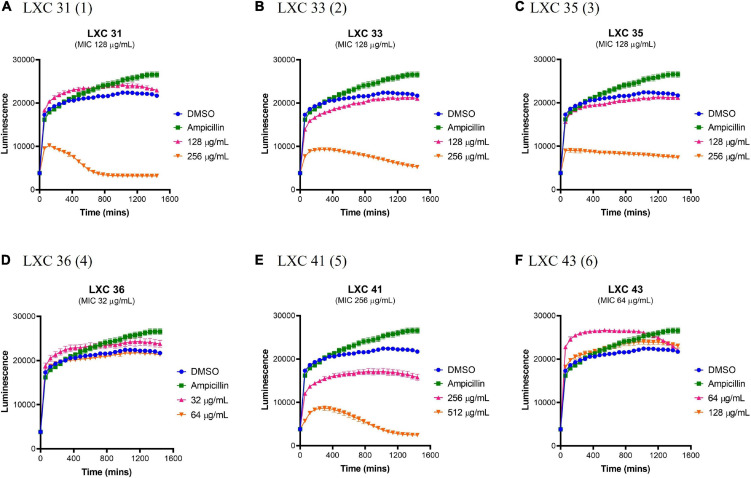
The LXC compounds are generally non-toxic at a concentration of 1 × MIC. **(A–F)** Real-time liver cell viability measurements for HepG2 after treatment with 1 × (pink line) and 2 × (brown line) MIC values. A 2.0% (v/v) DMSO (vehicle control; blue line) and 50 μg/mL ampicillin (green line) were used as controls. Cell viability was measured every 5 min for 24 h at 37°C and 5% CO_2_ on a Cytation5^®^ Cell Imaging Multi-Mode Reader using the RealTime-Glo™ MT Cell Viability Assay reagent. The results are presented in mean ± SEM (hourly SEM data was presented).

The effects of compounds **1** to **6** on the growth and metabolism of HepG2 ATCC HB-8065 mammalian liver cells were investigated using the RealTime-Glo™ MT Cell Viability Assay Kit (Figure 8). This photometric assay measured the ability of metabolically active cells to reduce the NanoLuc substrate to a luminescent product. Cells were treated over 24 h with either 1 × or 2 × MIC concentrations (pink curves and orange curves, respectively, Figure 8) and the resulting growth curves were compared against a vehicle only control of 2.0% (v/v) DMSO (blue curves, Figure 8). At 1 × MIC, the only compound that displayed cytotoxicity was compound **5** (Figure 8E). When the concentration of the compounds was increased to 2 × MIC, cytotoxicity was also observed for **1**, **2**, **3**. Importantly, compounds **4** and **6** were devoid of undesirable toxicity even at 2 × MIC.

**FIGURE 9:**
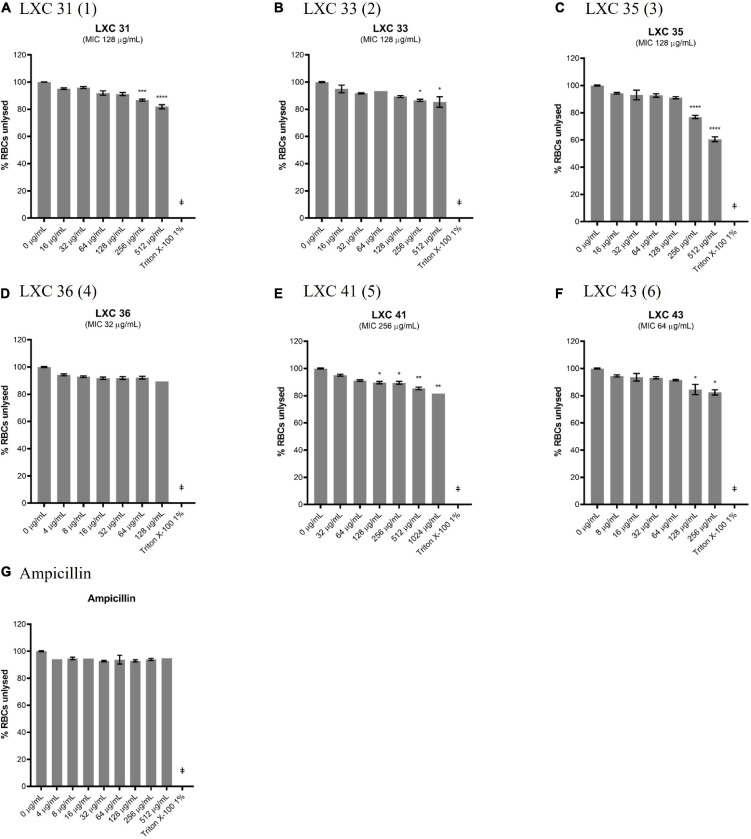
Most of the LXC compounds are not hemolytic up to 128 μg/mL. **(A–G)** Freshly washed human RBCs in PBS solution (137 mM NaCl, 2.7 mM KCl, 1.46 KH_2_PO_4_, 8.1 mM NaH_2_PO_4_, pH 7.4) was exposed to 8 μL of LXC compounds with concentrations ranging from 0 μg/mL up to 4× their MIC values in 2.0% (v/v) DMSO. A 1% (v/v) Triton X-100 was used to indicate complete RBC lysis (^‡^). Ampicillin (0–512 μg/mL) was used as a control or drug that does not cause RBC lysis. The assays were performed in quadruplicates. The plates were incubated at 37°C while constantly shaking at 100 rpm for 1 h. Intact RBCs were removed by centrifugation and the presence of hemolytic products in the supernatant were determined by measuring the absorbance at A_450_ nm. The results are presented in mean ± SEM. Statistical analysis was performed using one-way ANOVA and the asterisks (*) represent statistical significance (*p < 0.05; **p < 0.005; ***p < 0.0005; ****p < 0.0001).

The lack of cytotoxicity observed in the HepG2 cell viability assay above was also confirmed in a hemolytic assay. Here, fresh human red blood cells were incubated for 1 h with varying concentrations of compounds, after which the release of haem from lysed cells was measured spectroscopically (Figures 9A–F). The non-toxic antibiotic ampicillin served as a non-lytic control (Figure 9G). Treatment of cells with 1% (v/v) Triton X-100 was sufficient to completely disrupt the red blood cells and served as a control for lysis (Figure 9). None of the cinnamaldehyde derivatives showed hemolytic activity at 1 × MIC, except di-methoxy analog **5** which was only non-lytic at a low concentration (0.25 × MIC, Figure 9E). Noteworthy was the observation that cell lysis was observed at 2 × MIC for most compounds except for brominated analog **4** that was devoid of hemolytic activity even at the highest concentration tested (4 × MIC, Figure 9D). Together, the data from the hemolysis and HepG2 cytotoxicity assays help present halogenated analogs **4** and **6** as promising lead compounds.

**FIGURE 10:**
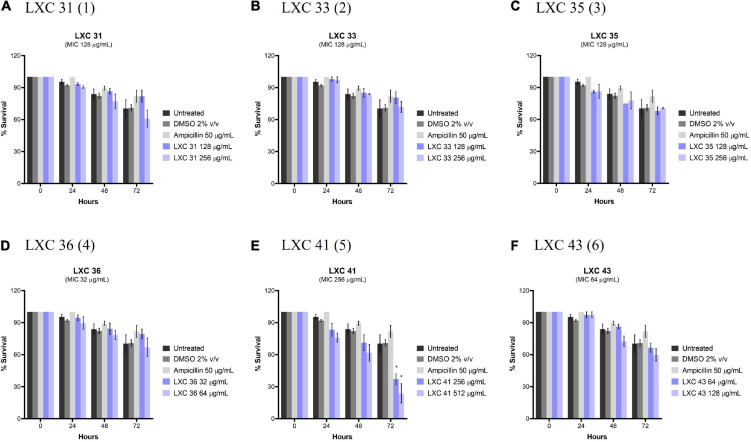
The LXC compounds (apart from compound **5**) were not cytotoxic to *C. elegans* nematodes. **(A–F)** *C. elegans* nematodes were cultured on nematode growth media agar plate and harvested in optimized growth media, with *E. coli* as its primary source of nutrient. Newly harvested adult nematodes were investigated for toxicity in the presence of the LXC compounds at 1× and 2× their MIC values for a timespan of up to 72 h. The nematodes were counted under a light microscope at 400× magnification and the live nematodes at 72 h was indicated as a fraction of the starting number of nematodes (percentage survival). The results are presented in mean percentage of survival ± SEM. Statistical analysis was performed using two-way ANOVA and the asterisks (*) represent statistical significance: LXC 41 (**5**) at time 72 h (*p < 0.05).

Potential *in vivo* toxicity of the LXC compounds was investigated using *C. elegans* (Figure 10). The nematodes were treated with the LXC compounds at 1 × and 2 × their MIC values (dark and light purple bars, respectively, Figure 10) for up to 3 days. Live nematodes, which are readily distinguished from dead organisms by their physical appearance under the microscope (Chai et al., 2020), were enumerated every 24 h. The survival rates were compared to untreated controls (black bars, Figure 10), a vehicle only control (2% (v/v) DMSO, dark gray bars, Figure 10) and the non-toxic antibiotic ampicillin (light gray bars, Figure 10). Encouragingly, none of the compounds in this series displayed toxicity toward *C. elegans*, except for di-methoxy analog **5** which showed significant toxicity (*p* < 0.05) but only after 72 h treatment (Figure 10E). This result agreed with that seen with the cytotoxicity in mammalian liver cells (Figure 8). Compounds **1**, **2** and **3** displayed toxicity against mammalian liver cells at 2 × MIC (Figure 8) yet the nematode data suggests these compounds do not have *in vivo* toxicity in this model at 2 × MICs for up to 72 h (Figure 10).

## Conclusion

This study explored the antimicrobial potency, on-target activity, and pre-clinical suitability of a panel of 2-methylbenzimidazoyl cinnamaldehyde analogs. In our previous study, cinnamaldehyde derivatives **1** to **6** were identified with antimicrobial activity against methicillin-sensitive *S. aureus* and modest activity against *E. coli* (Li et al., 2015a). In the current study, antimicrobial sensitivity screening was expanded using an extended panel of Gram-negative pathogens. Results from this screen revealed the novel observation that **1** to **6** possessed antimicrobial activity against *A. baumannii*. This finding is most welcome in the fight to combat AMR, especially against this clinically important and difficult to treat pathogen. The antimicrobial activity of the LXC compounds against an XDR strain of *A. baumannii* is timely and of great significance as *A. baumannii* is on top of the WHO list of critically important organisms in need of urgent drug discovery (World Health Organization, 2017). The potency of all six compounds was increased when the efflux pump inhibitor, PAβN, was added to the assay. The synergistic activity observed for the combination treatment for the XDR *A. baumannii* strain that is otherwise only responsive to the last resort antibiotic colistin is of particular significance.

The thorough biological characterization of cinnamaldehyde derivatives **1** to **6** presented here identified the di-chlorinated derivative **6** as a promising pre-clinical candidate for further chemical development. This compound had the requisite activities necessary in a lead compound, including antimicrobial potency, confirmation of on-target activity against FtsZ protein, lack of activity against the tubulin counter-target and good *in vitro* and *in vivo* safety profiles. Mono-brominated **4** should also be considered for further study and optimization. Whilst this compound showed undesirable inhibitory activity against tubulin *in vitro*, it was well-tolerated in both cell-based cytotoxicity assays as well as the nematode model. Interestingly, halogenated derivatives **2**, **3**, **4,** and **6** generally showed superior activity in biological testing relative to the non-substituted parent **1** compound. Together, the findings from this study highlight the potential of halogenated cinnamaldehyde derivatives as a new class of antibacterial that target FtsZ to combat clinically important bacterial pathogens, such as *A. baumannii*. This work also emphasizes previous literature that suggests that chemical halogenation is a promising route to improve the antibacterial activity of compounds in early stage antibacterial discovery (Domagala, 1994; Brighty and Gootz, 1997; Paparella et al., 2018).

## Data availability statement

The original contributions presented in this study are included in the article/Supplementary material, further inquiries can be directed to the corresponding author.

## Author contributions

HV, SS, and SM: design and concept. WC, JW, SP, AO, CD, KF, and XL: experimental. WC, JW, SP, SS, MS, and HV: writing. All authors have approved the final article.
