# Supplementary material for: Cinnamaldehyde derivatives act as antimicrobial agents against Acinetobacter baumannii through the inhibition of cell division
Source: Front Microbiol. 2022 Aug 29;13:967949. doi: 10.3389/fmicb.2022.967949 (PMC9465178; doi:10.3389/fmicb.2022.967949)
Supplement: Supplementary file 1 [file Data_Sheet_1.docx]

Supplementary Material

Cinnamaldehyde derivatives act as antimicrobial agents against *Acinetobacter baumannii* through the inhibition of cell division

Wern Chern Chai^1^, Jonathan J Whittall^1^, Steven W Polyak^1^, Klyie Foo^1^, Xin Li^2^, Cameron J Dutschke^1^, Abiodun D Ogunniyi^3^, Shutao Ma^2^, Matthew J Sykes^1^, Susan J Semple^1,4^, Henrietta Venter^1*^

^1^Health and Biomedical Innovation, Clinical and Health Sciences, University of South Australia, Adelaide 5000, South Australia, Australia

^2^Department of Medicinal Chemistry, Key Laboratory of Chemical Biology (Ministry of Education), School of Pharmaceutical Sciences, Cheeloo College of Medicine, Shandong University, Jinan 205512, P.R. China

^3^Australia Centre for Antimicrobial Resistance Ecology, School of Animal and Veterinary Sciences, University of Adelaide, Roseworthy Campus, Roseworthy 5371, South Australia, Australia

^4^Quality Use of Medicines and Pharmacy Research Centre, Clinical and Health Sciences, University of South Australia, Adelaide 5000, South Australia, Australia

***Correspondence:**Henrietta Venter
Rietie.Venter@unisa.edu.au

Keywords:
Antimicrobial resistance, Antimicrobial drug development, FtsZ, FtsZ inhibitor, Cinnamaldehyde, Gram-negative, XDR *Acinetobacter baumannii*

**Table S1.** Antibacterial activity of the LXC compounds on ESKAPE pathogens and other microorganisms tested in this study.

| **Strains** | OXA | LEF | RIF | LXC Compounds | | | | | |
| --- | --- | --- | --- | --- | --- | --- | --- | --- | --- |
|  |  |  |  | 31 | 33 | 35 | 36 | 41 | 43 |
| *Enterobacter aerogenes* ATCC 13408 | >16 | 1 | 16 | >256 | >256 | >256 | >256 | >256 | >256 |
| *Escherichia coli* ATCC 35218 | >64 | 0.004 | >16 | >256 | >256 | >256 | >256 | >256 | >256 |
| *Escherichia coli* WT BW 25113 | >64 | 0.25 | >16 | >256 | >256 | >256 | >256 | >256 | >256 |
| *Klebsiella pneumoniae* ATCC 4352 | >64 | 1 | >64 | >256 | >256 | >256 | >256 | >256 | >256 |
| *Klebsiella pneumoniae* ATCC 13883 | >64 | 1 | 8 | >256 | >256 | >256 | >256 | >256 | >256 |
| *Klebsiella pneumoniae* ATCC 33495 | >64 | 1 | 32 | >256 | >256 | >256 | >256 | >256 | >256 |
| Methicillin resistant *Staphylococcus aureus* | 16 | 1 | 32 | 256 | >256 | >256 | >256 | >256 | 64 |
| *Pseudomonas aeruginosa* WT PAO1 | >64 | 0.5 | 32 | 256 | 256 | >256 | >256 | >256 | 256 |
| *Pseudomonas aeruginosa* ATCC 27853 | >64 | 1 | 32 | >256 | >256 | >256 | 256 | >256 | >256 |
| *Streptococcus pneumoniae* (type 3, mucoid strain) ATCC 6303 | n.t | 1 | n.t. | 256 | 256 | 256 | 256 | 256 | 256 |
| *Streptococcus pyogenes* ATCC 10389 | n.t. | 1 | n.t. | >256 | >256 | >256 | >256 | >256 | >256 |

Legend:

OXA oxacillin LEF levofloxacin RIF rifampicin

n.t. not tested

**Table S2.** Calculated binding energies from *in silico* docking.

| **PDB** | **3VOB** | **5XDT** | **5XDU** | **6KVP** |
| --- | --- | --- | --- | --- |
| **Ligand** | PC190723 | TXA707 | TXA6101 | CH3-TXA6101 |
| **PC190723** | -14.88 | -12.77 | -14.26 | -14.68 |
| **TXA707** | -12.48 | -13.16 | -15.21 | -15.88 |
| **TXA6101** | -11.17 | -13.00 | -18.86 | -16.96 |
| **LXC 31 (1)** | -12.10 | -14.44 | -15.44 | -15.13 |
| **LXC 33 (2)** | -12.29 | -15.08 | -15.60 | -15.05 |
| **LXC 35 (3)** | -11.02 | -14.84 | -15.99 | -15.36 |
| **LXC 36 (4)** | -11.96 | -15.10 | -15.59 | -15.63 |
| **LXC 41 (5)** | -11.41 | -14.18 | -14.62 | -14.20 |
| **LXC 43 (6)** | -11.84 | -14.48 | -15.90 | -14.84 |

*In silico* docking studies were performed using OpenEye Scientific Software (McGann 2012). Molecular binding of LXC compounds **1** to **6** to FtsZ was analysed alongside ligands PC190723 (PDB 3VOB) (Matsui et al. 2012), TXA 707 (PDB 5XDT) (Fujita et al. 2017), TXA 6101 (PDB 5XDU) (Fujita et al. 2017), and a methylated analogue thereof (PDB 6KVP) (Ferrer-González et al. 2017). Compounds **1** to **6** were all predicted to bind with comparable affinities as the *bona fide* benzamide derivatives.

| **(A)**  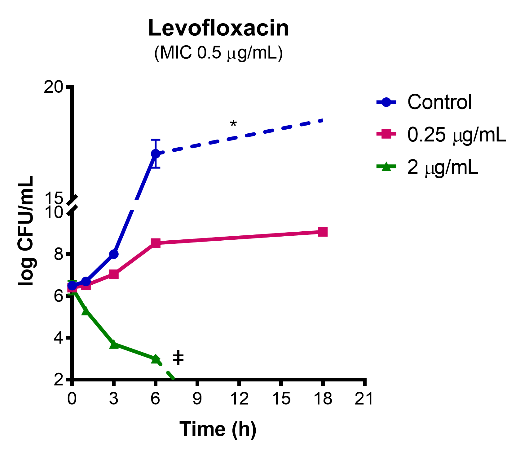 | **(B)**  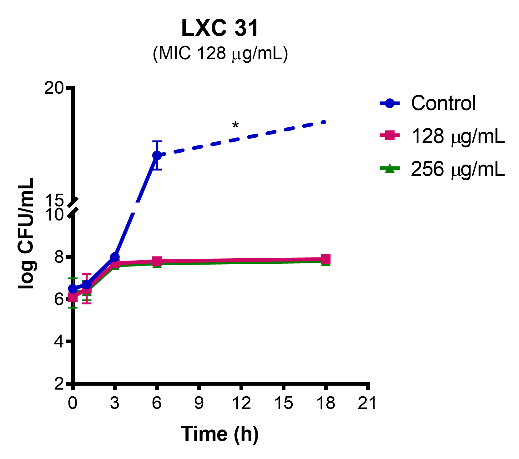 | **(C)**  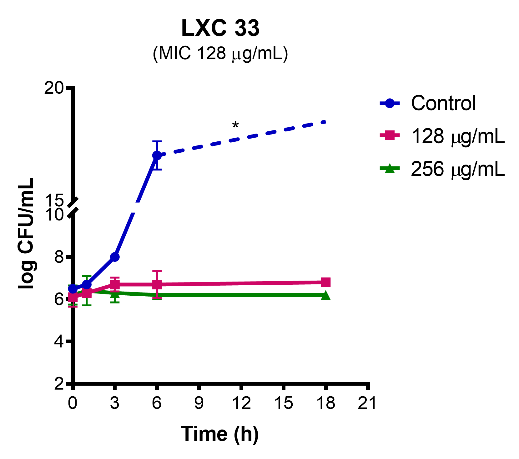 |
| --- | --- | --- |
| **(D)**  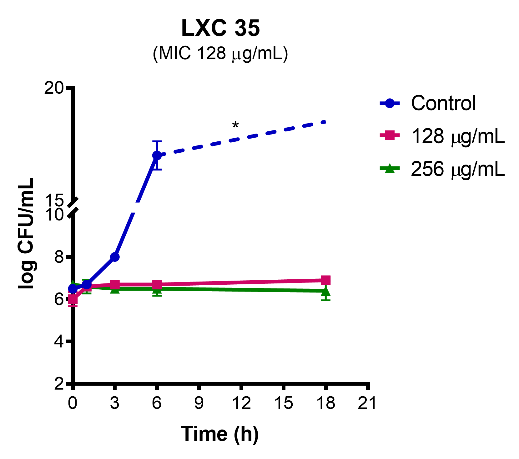 | **(E)**  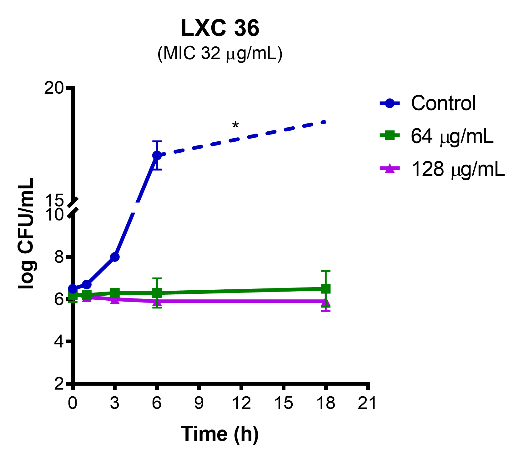 | **(F)**  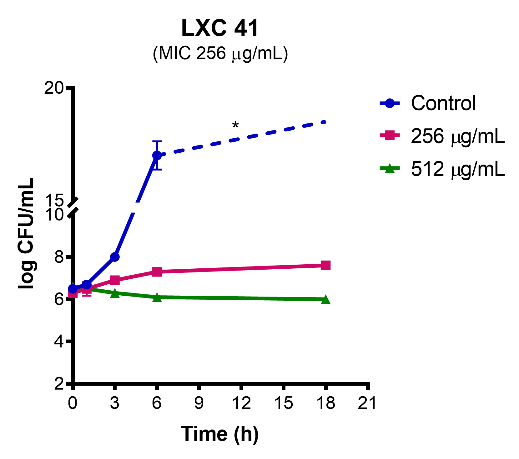 |
| **(G)**  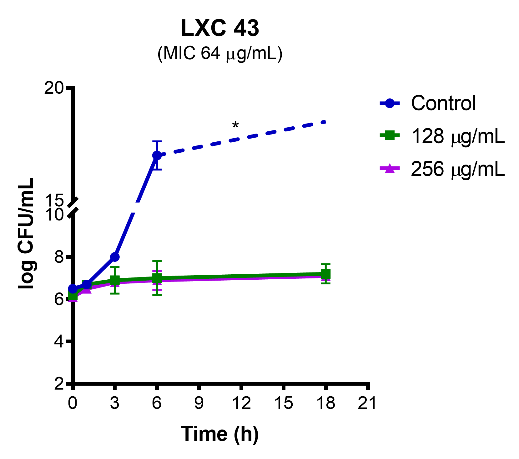 |  |  |

**Figure S1.** Time-kill curves for LXC compounds indicate a bacteriostatic mechanism of action.

Viable cell counts were performed for (A) *A. baumannii* ATCC 19606 in the absence of levofloxacin (blue line) and in the presence of levofloxacin at bacteriostatic (0.25 µg/mL) and bactericidal (2 µg/mL) concentrations. (B-G) *A. baumannii* was tested in the absence of the LXC compounds in 2.5% (v/v) DMSO (blue line), 1× MIC (pink line) and 2× MIC (green line) or 4× MIC (purple line). The results are representative of three independent experiments. The results are presented as mean log CFU/mL ± SEM. The asterisk (*) represents viable cell counts greater than 1.7×10^10^ CFU/mL and the alveolar (ǂ) represents viable cell counts below 1×10^3^ CFU/mL.


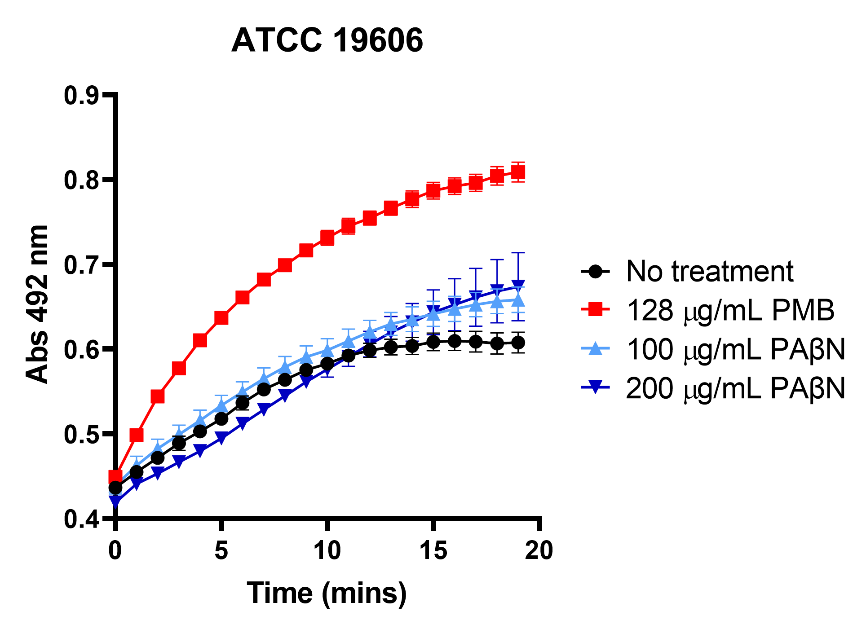


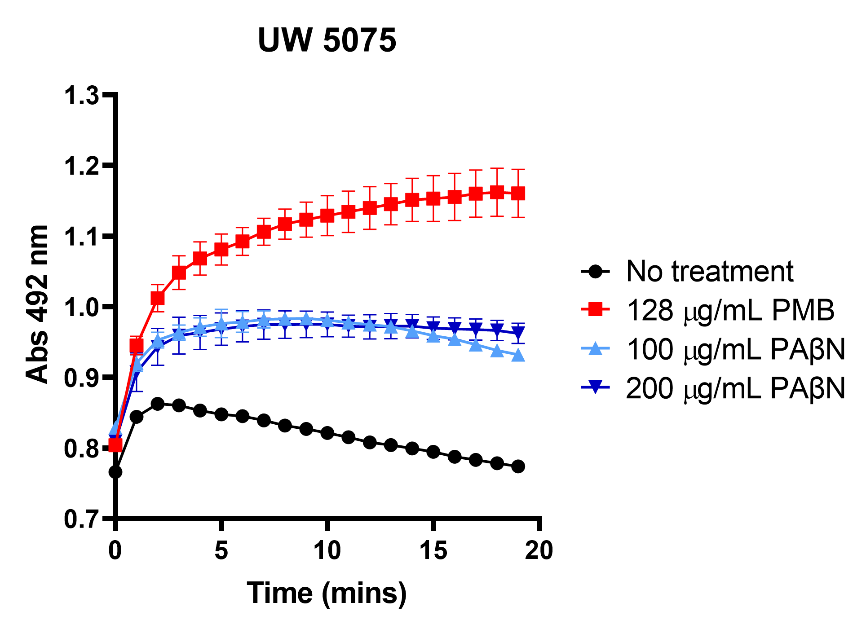


**Figure S2.** PAβN does not damage the bacterial outer membrane.

*A. baumannii* ATCC 19606 and the extensively resistant strain UW 5075 were assayed for permeabilisation of the outer membrane by the efflux pump inhibitor PAβN. Nitrocefin was added to the cells that received no treatment (black circles), cells treated with the outer membrane permeabilizer polymyxin B (PMB, red squares) or PAβN at 100 µg/mL (light blue triangles) or 200 µg/mL (dark blue triangles). Nitrocefin hydrolysis by the periplasmic β-lactamase was observed as an increase in absorbance at 490 nm. Representative traces are shown from triplicate experiments performed on different days. Symbols represent the mean of triplicate readings ± SEM.

Time (h)

0 1 3 6 18

*A. baumannii
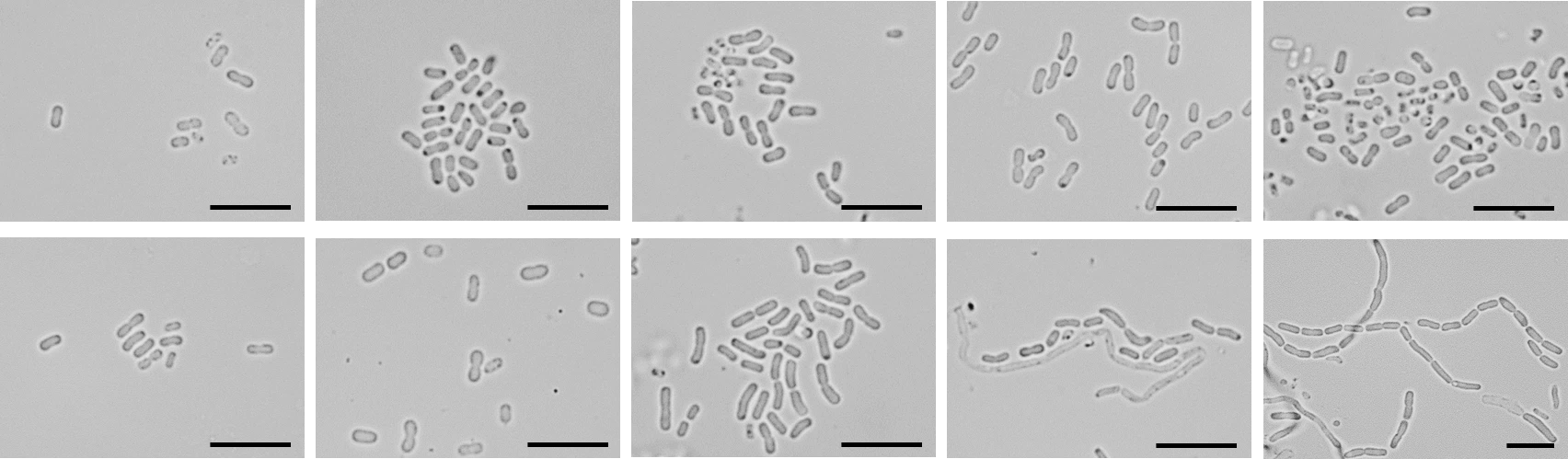
*

ATCC 19606

Levofloxacin

0.125 µg/mL


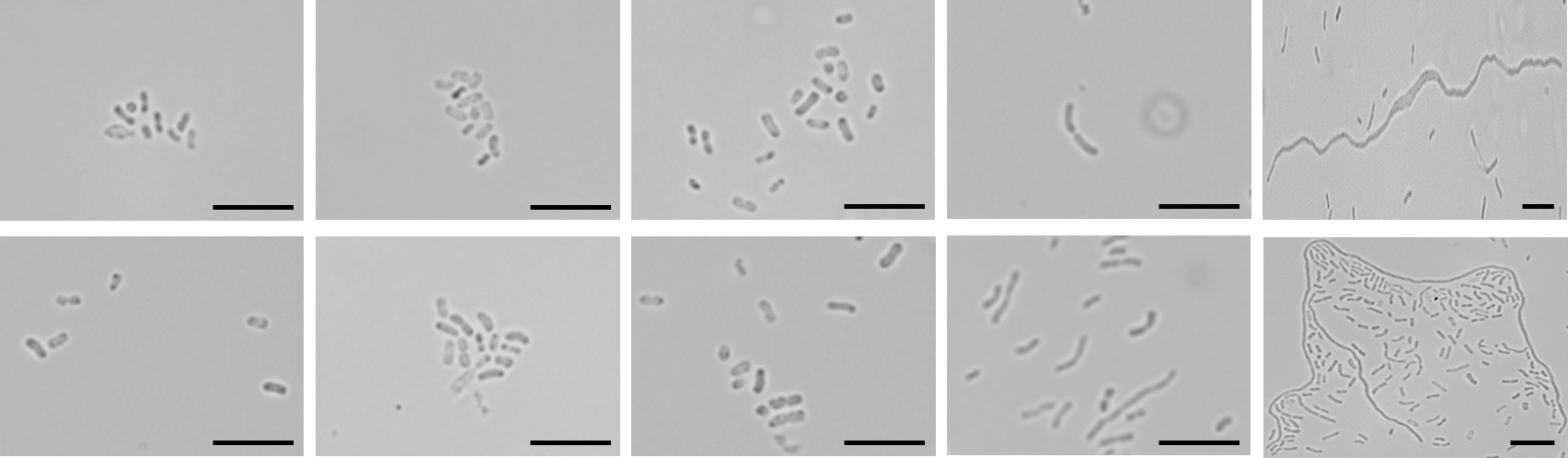


Divin

32 µg/mL

Divin

64 µg/mL


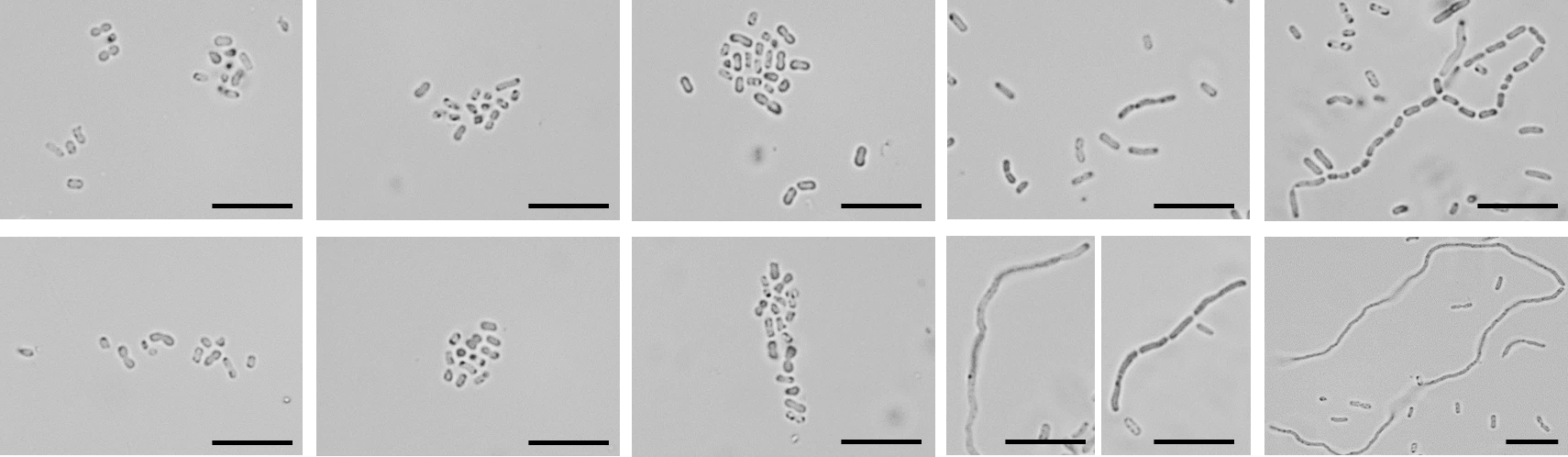
LXC 31

128 µg/mL

LXC 31

256 µg/mL


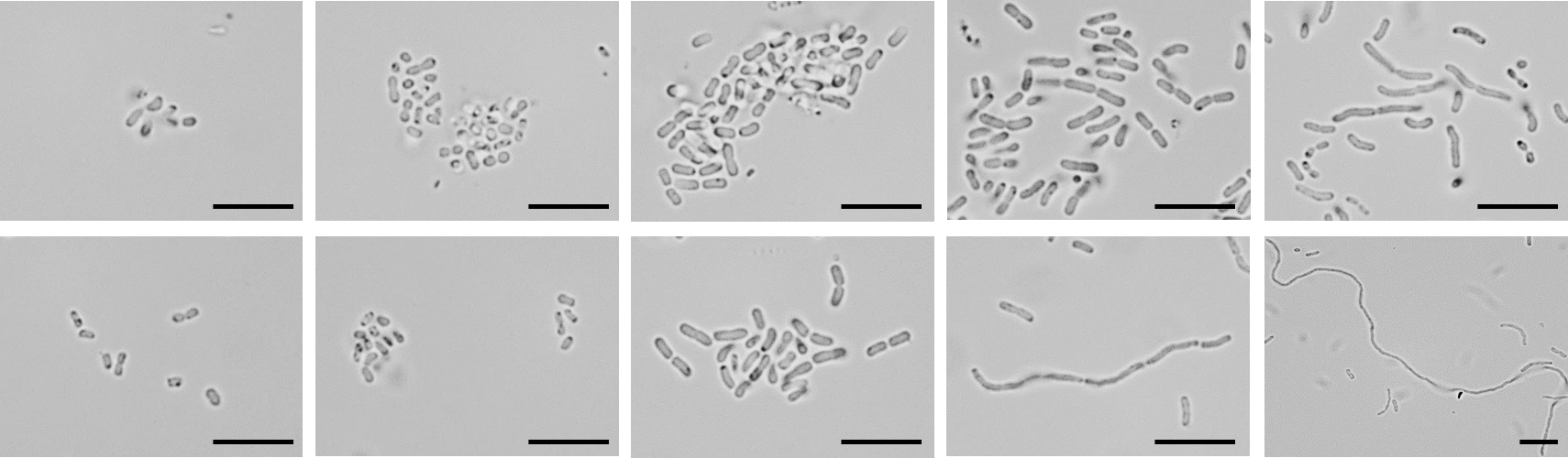


LXC 33

128 µg/mL

LXC 33

256 µg/mL

Time (h)

0 1 3 6 18


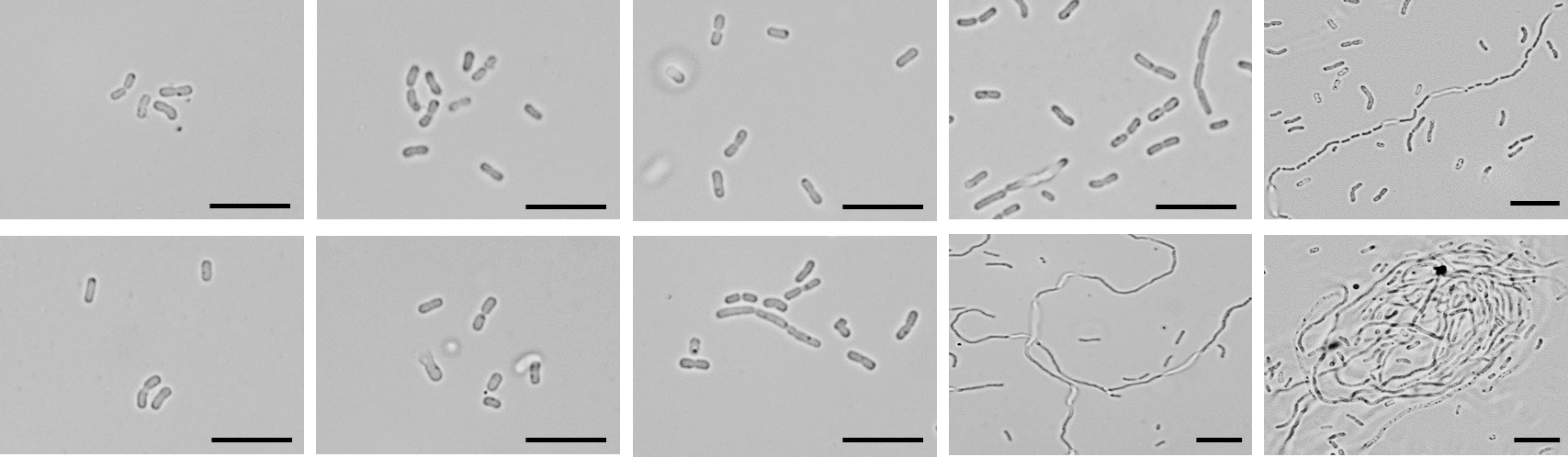
LXC 35

128 µg/mL

LXC 35

256 µg/mL


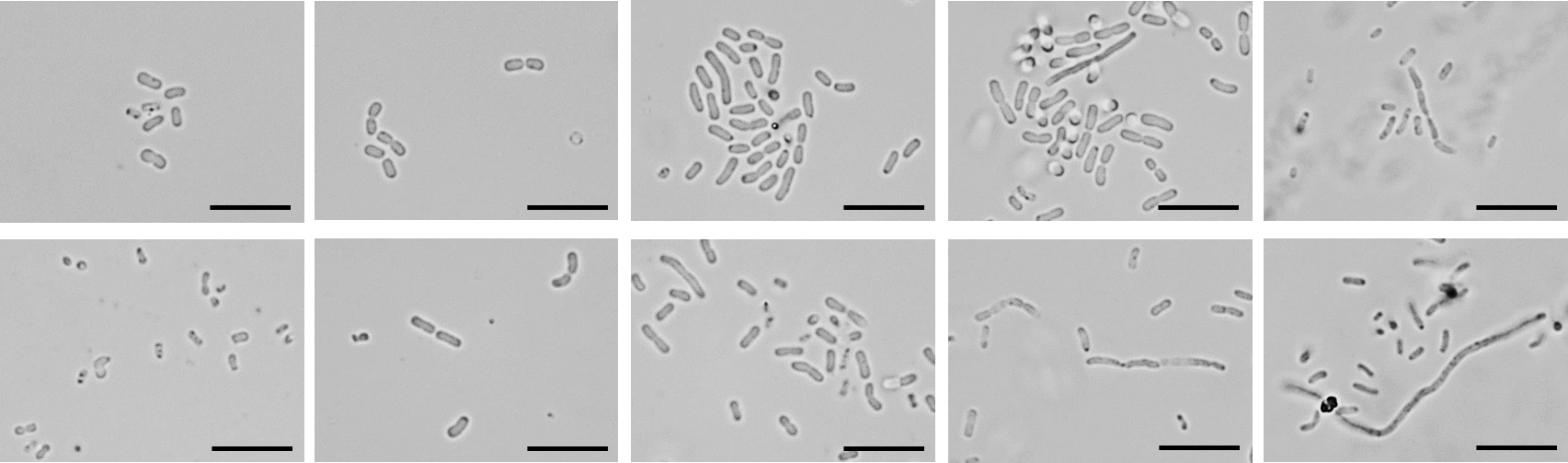


LXC 36

64 µg/mL

LXC 36

128 µg/mL


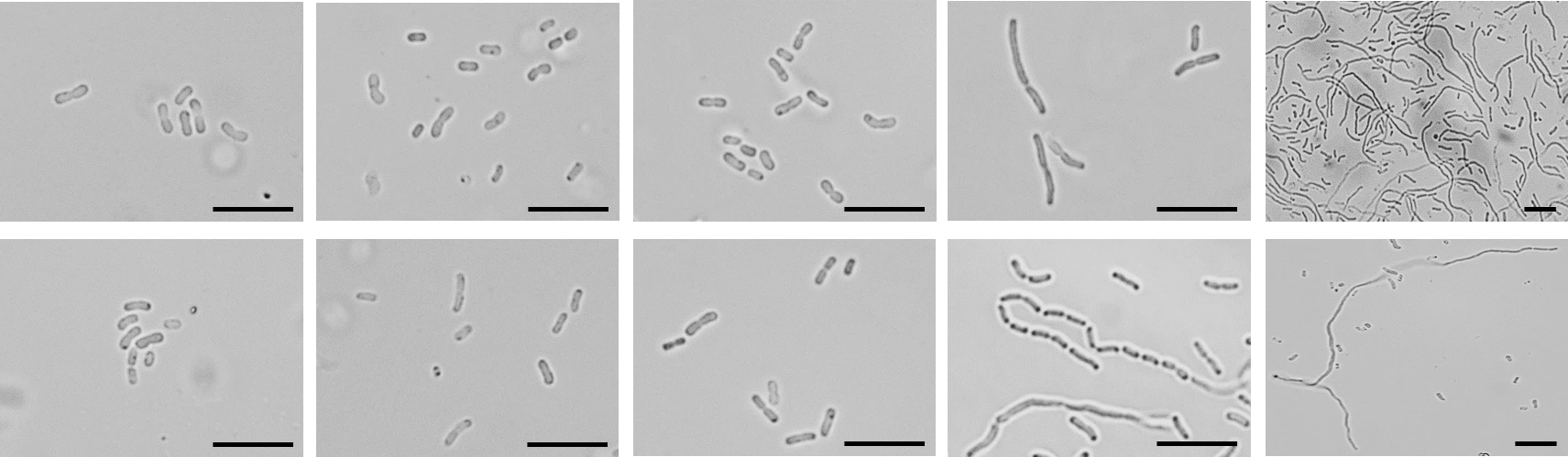


LXC 41

256 µg/mL

LXC 41

512 µg/mL


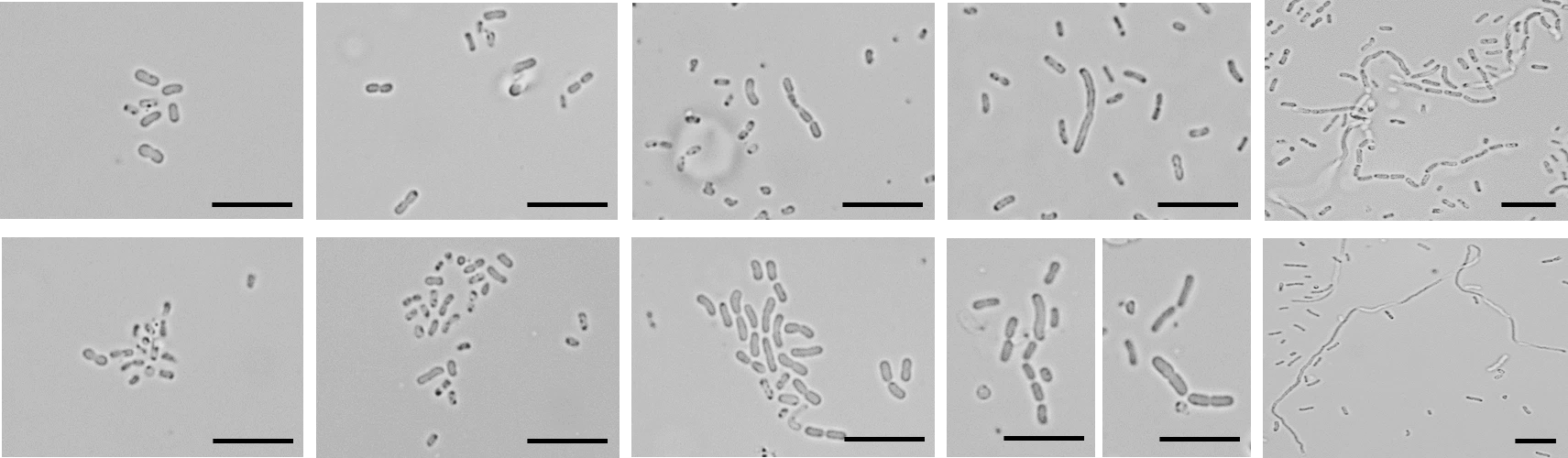


LXC 43

128 µg/mL

LXC 43

256 µg/mL

**Figure S3.** LXC compounds were tested on *A. baumannii* ATCC 19606 to determine the phenotypic changes.

Four of the compounds (LXC 31, 33, 35 and 41) were tested at 1× and 2× their minimum inhibitory concentration (MIC) whereas LXC 36 and 43 were tested at 2× and 4× MIC. The phenotypic changes in *A. baumannii* morphology were analysed under a light microscope (100× magnification) at time 0, 1, 3, 6 and 18 h. Scale bar is 50 µm.

| LXC 31 (**1**)  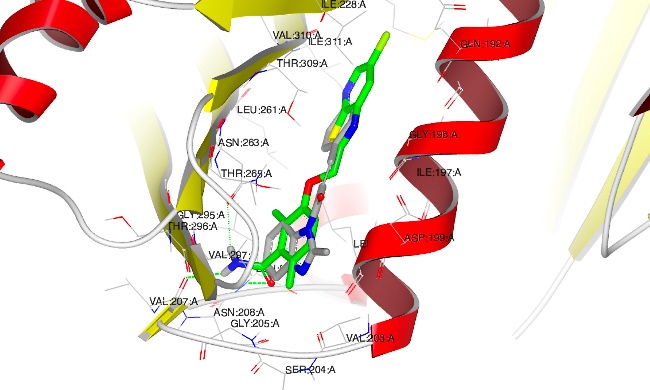 | LXC 33 (**2**)  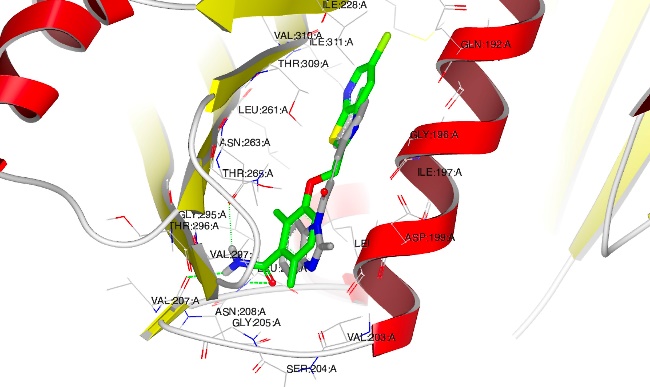 |
| --- | --- |
| LXC 35 (**3**)  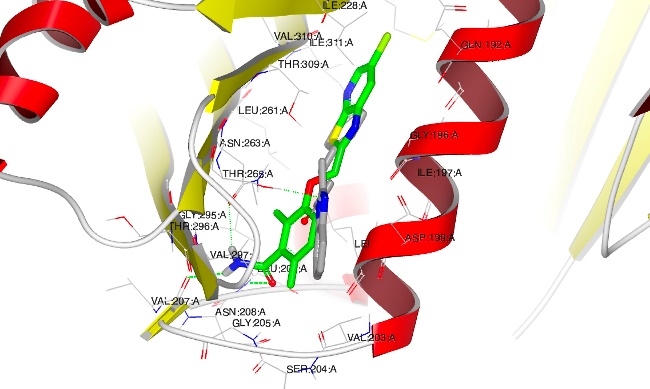 | LXC 36 (**4**)  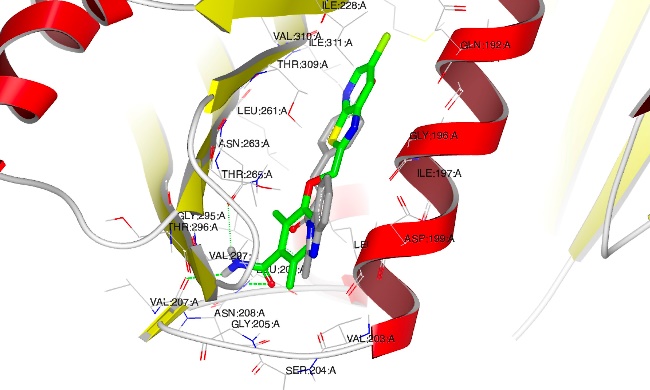 |
| LXC 41 (**4**)  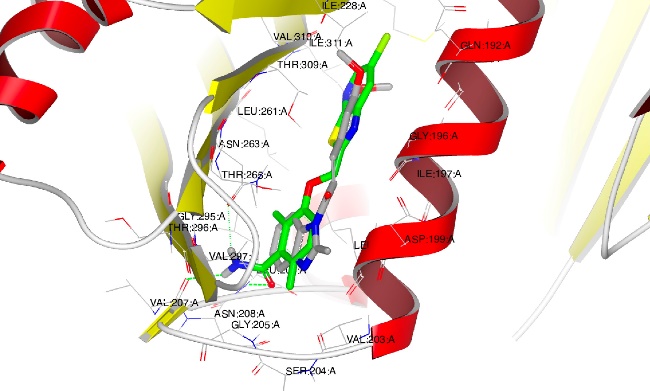 | LXC 43 (**6**)  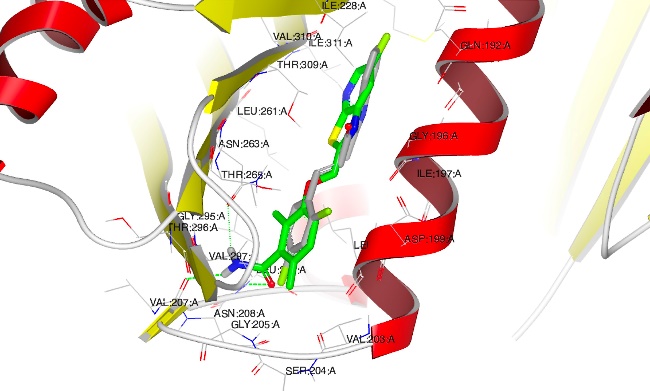 |

**Figure S4.** Molecular analysis of FtsZ binding cinnamaldehyde analogues **1** to **6**.

*In silico* docking studies were performed using OpenEye Scientific Software (McGann 2012). Molecular binding of LXC compounds **1** to **6** (grey wire) are shown above alongside *bona fide* FtsZ inhibitor PC190723 (green wire; PDB 3VOB) (Matsui et al. 2012).

**References**

Ferrer-González, E, Kaul, M, Parhi, AK, LaVoie, EJ & Pilch, DS 2017, 'β-lactam antibiotics with a high affinity for PBP2 act synergistically with the FtsZ-targeting agent TXA707 against methicillin-resistant *Staphylococcus aureus*', *Antimicrob. Agents Chemother.,* vol*.* 61, no. 9, pp. e00863-00817.

Fujita, J, Maeda, Y, Mizohata, E, Inoue, T, Kaul, M, Parhi, AK, Lavoie, EJ, Pilch, DS & Matsumura, H 2017, 'Structural flexibility of an inhibitor overcomes drug resistance mutations in *Staphylococcus aureus* FtsZ', *ACS Chemical Biology,* vol*.* 12, no. 7, pp. 1947-1955.

Matsui, T, Yamane, J, Mogi, N, Yamaguchi, H, Takemoto, H, Yao, M & Tanaka, I 2012, 'Structural reorganization of the bacterial cell-division protein FtsZ from *Staphylococcus aureus*', *Acta Crystallogr., Sect. D: Biol. Crystallogr.,* vol*.* 68, no. 9, pp. 1175-1188.

McGann, M 2012, 'FRED and HYBRID docking performance on standardized datasets', *J Comput Aided Mol Des,* vol*.* 26, no. 8, pp. 897-906.
